# Quinoline/thiazole compounds as selective acetylcholinesterase inhibitors: synthesis and biological assessment

**DOI:** 10.1039/d6md00165c

**Published:** 2026-04-07

**Authors:** Berkant Kurban, Derya Osmaniye, Serkan Levent, Yusuf Özkay, Zafer Asım Kaplancıklı

**Affiliations:** a Department of Pharmaceutical Chemistry, Faculty of Pharmacy, Afyonkarahisar Health Sciences University 03030 Afyonkarahisar Türkiye; b Graduate School, Anadolu University 26470 Eskişehir Türkiye; c Department of Pharmaceutical Chemistry, Faculty of Pharmacy, Anadolu University 26470 Eskişehir Türkiye zakaplan@anadolu.edu.tr; d Central Research Laboratory (MERLAB), Faculty of Pharmacy, Anadolu University 26470 Eskişehir Türkiye; e Department of Analytical Chemistry, Faculty of Pharmacy, Anadolu University 26470 Eskişehir Türkiye; f Pharmacy Services, Vocational School of Health Services, Bilecik Şeyh Edebali University 11000 Bilecik Türkiye

## Abstract

Acetylcholine (ACh), acetylcholinesterase enzyme (AChE), and AChE inhibition are of great importance in the treatment of neurodegenerative diseases. When it comes to AChE inhibition, FDA-approved AChE inhibitors such as donepezil are actively used in the treatment of neurodegenerative disorders. However, there is still a need for novel and unique inhibitors for the radical treatment of these diseases. The aim of this study was to design and evaluate new inhibitors for the treatment of neurodegenerative diseases. To meet this need, a series of new quinoline/thiazole derivative compounds were designed and synthesized, and their structures were elucidated using ^1^H-NMR, ^13^C-NMR and HRMS. There are many studies showing that quinolines and thiazoles have high potential efficacy in AChE inhibition. The compounds were synthesized through a multi-step synthetic route, and their inhibitory activities were subsequently examined using. Compound 3i emerged as the most promising derivative, distinguished by its interactions with amino acids such as Trp86 and Trp286 in *in silico* studies, and its potent *in vitro* activity against AChE with an IC_50_ value of 0.027 ± 0.002 μM. These findings suggest that these novel quinoline/thiazole derivatives could be potential candidates for the development of new therapies for neurodegenerative diseases.

## Abstract 2

Acetylcholine (ACh), acetylcholinesterase enzyme (AChE), and AChE inhibition are of great importance in the treatment of neurodegenerative diseases.

## Graphical abstract

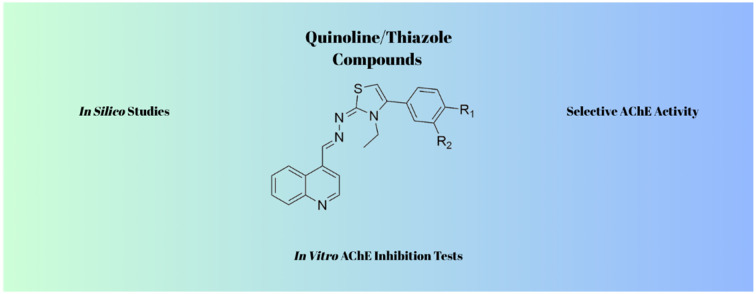

## Introduction

Alzheimer's disease (AD) is the most commonly observed neurodegenerative disorder today. Moreover, AD ranks among the leading causes of mortality alongside cancer and cardiovascular diseases. Alzheimer's disease primarily affects cognitive functions such as speech and thinking. In 1901, Alois Alzheimer identified the disease in a 51-year-old female patient, and in 1906, he defined it, leading to its recognition in the literature as Alzheimer's disease.^1–4^

Several hypotheses have been proposed for the treatment of Alzheimer's disease (AD); among which, the cholinergic hypothesis is one of the most established. This hypothesis is based on the preservation of acetylcholine levels in the postsynaptic cleft. In line with this hypothesis, compounds that inhibit the enzyme acetylcholinesterase (AChE) have long been utilized in the treatment of AD. Donepezil, rivastigmine, tacrine, and galantamine are among the active pharmaceutical ingredients developed based on this approach. Despite their decades-long use and relevance in AD therapy, these compounds have shown limited efficacy and exhibit undesirable side effect profiles, highlighting the need for novel and more effective alternatives. In response to this therapeutic gap, a series of novel AChE inhibitors containing thiazole/quinoline scaffolds have been designed, synthesized, and evaluated through *in silico* and *in vitro* activity assays.^5–9^

The primary function of cholinesterase (ChE) enzymes is the hydrolysis of acetylcholine (Ach). ChE enzymes are classified into two types: acetylcholinesterase (AChE, EC 3.1.1.7) and butyrylcholinesterase (BChE, EC 3.1.1.8). AChE plays a critical role in the pathophysiology of neurodegenerative diseases due to its interaction with ACh as the substrate, the observation of reduced AChE levels in conditions such as Alzheimer's disease, and its association with pathological protein adhesion, oxidative stress, apoptosis, *etc.* Abnormalities in cholinergic neurotransmission are significant in the progression of Alzheimer's disease, and thus, ChE inhibitors such as donepezil, galantamine, rivastigmine, tacrine, and huperzine A have been used in AD treatment.^10–14^

Recent studies have focused on achieving AChE inhibition by targeting the π–π interactions between the quinoline ring and the peripheral anionic site (PAS) of the enzyme. However, in the present study, the design strategy was altered such that the quinoline moiety was intended to interact with the catalytically active site (CAS) of AChE, while substituted benzene structures were aimed at interacting with the PAS region. Additionally, the hydrazinylidene-thiazole scaffold was designed to function as a linker between these two pharmacophores.^15–17^

The thiazole scaffold has attracted significant attention in medicinal chemistry due to its broad spectrum of biological activities. In the context of AD treatment, this moiety is particularly valuable as it can establish critical hydrogen bonds and hydrophobic interactions with key amino acid residues within the AChE active site, thereby enhancing binding affinity.^18–21^

Studies have shown that the quinoline ring plays an important role in AChE inhibition. *In silico* studies demonstrate that the quinoline ring interacts favorably with the tryptophan 86 (TRP86) amino acid in the cationic active site of AChE. Since the TRP86 interaction is also observed in FDA-approved AChE inhibitors such as donepezil, quinoline-based compounds were preferred in the design phase. Furthermore, the literature includes compounds containing both quinoline and thiazole rings, which possess high AChE inhibition potential.^22–24^

## Results and discussion

### Chemistry

*N*-Ethylhydrazinecarbothioamide (1) was obtained by reacting excess ethyl isothiocyanate with hydrazine hydrate. After the obtained *N*-ethylhydrazinecarbothioamide (1) was crystallized from ethanol, it was refluxed with quinoline-4-carbaldehyde in absolute ethanol. The reaction, monitored by thin-layer chromatography (TLC), was completed by filtering the precipitated product. The product was then crystallized from ethanol, yielding *N*-ethyl-2-(quinolin-4-ylmethylene)hydrazine-1-carbothioamide (2).

**Scheme 1:**
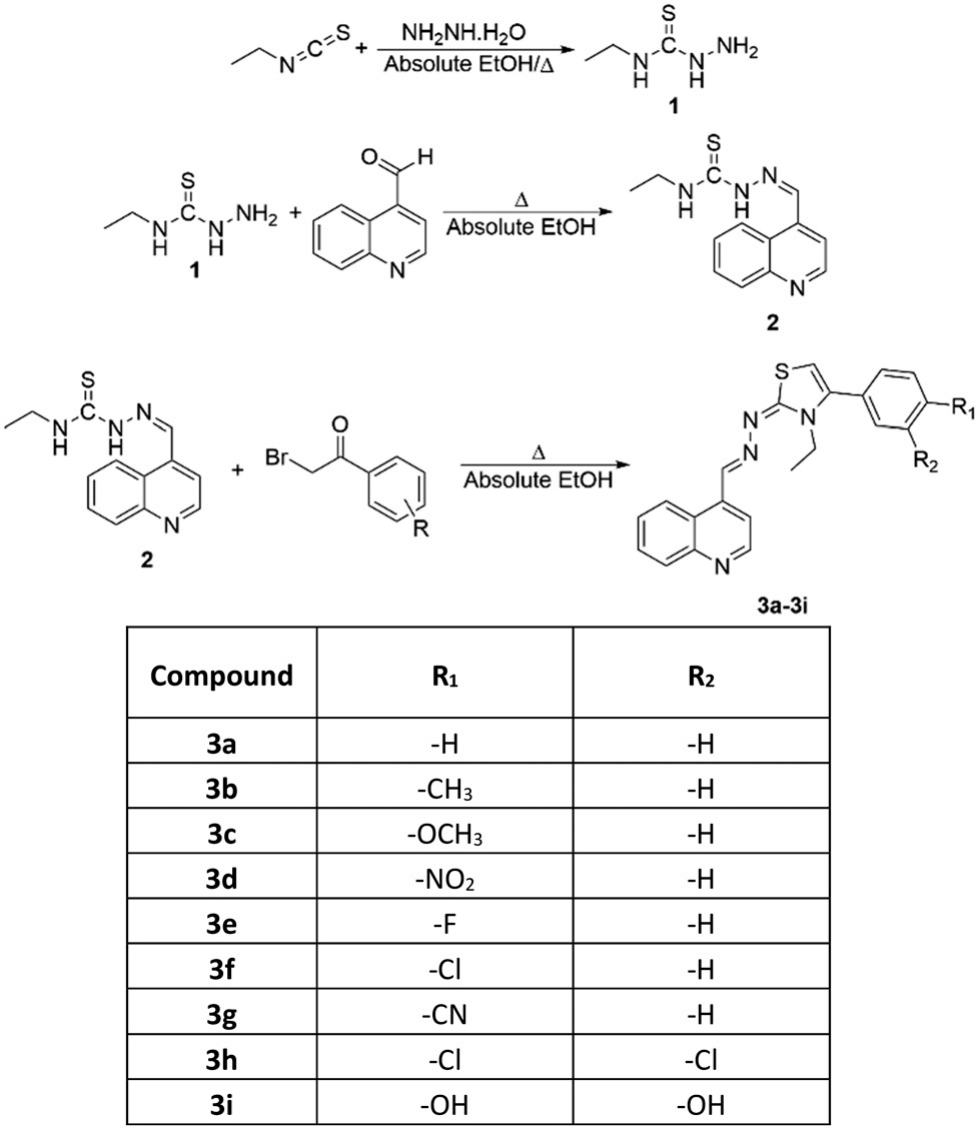
Synthesis pathway for the obtained compounds (3a–3i).

In the third and final step, *N*-ethyl-2-(quinolin-4-ylmethylene)hydrazine-1-carbothioamide (2) was refluxed in absolute ethanol with appropriate phenacyl bromide derivatives. The reaction was monitored by TLC, and the precipitated products were separated by filtration and crystallized from ethanol. In this way, as indicated in Scheme 1, nine different quinoline/thiazole derivative compounds were obtained.

Prior to conducting a two-dimensional correlation analysis to determine the complete proton and carbon assignments of compound 3h, the chemical shift values in the ^1^H and ^13^C NMR spectra were used to identify the positions of protons 10 and 11 and their corresponding carbons. Additionally, the coupling constants (*J*) and signal splitting patterns allowed for the identification of hydrogens 3, 5, and 6. The carbons bonded to these protons were unambiguously assigned using HSQC experiments. Furthermore, the HSQC results facilitated the correlation of protons 8, 12, 14, 15, 18, 19, 20, and 21 with their respective carbons, though their exact positions were confirmed *via* HMBC analysis. The proposed molecular structure was also found to be consistent with the NOESY results (Table 1).

**Table 1:** 2D ^1^H-NMR and ^13^C-NMR analyses of compound 3h

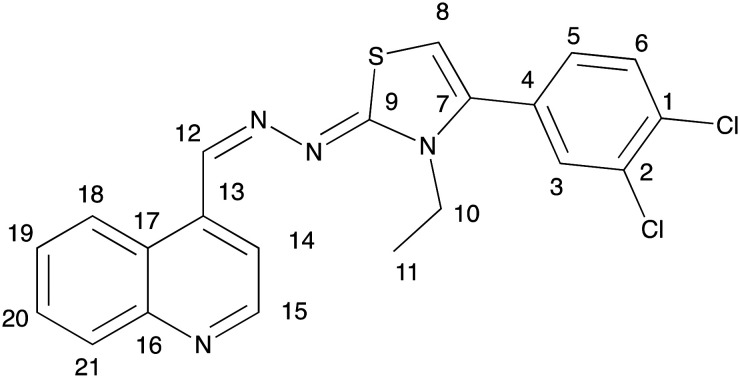		
# of atom	^1^H NMR value (ppm)	^13^C value (ppm)
1	—	132.2
2	—	133.1
3	7.91	131.7
4	—	130.8
5	7.60	130.0
6	7.84	131.6
7	—	138.9
8	6.95	106.2
9	—	174.4
10	3.99	42.0
11	1.19	14.0
12	9.10	143.7
13	—	139.7
14	8.29	118.1
15	9.16	144.2
16	—	147.9
17	—	125.4
18	7.94	129.9
19	8.25	122.3
20	8.13	134.3
21	9.07	126.5

### Anticholinesterase enzyme activity studies

Anticholinesterase enzyme activity studies were conducted to evaluate the inhibitory potential of the synthesized compounds (Table 2). Upon analysis of the results, compounds 3h and 3i stood out due to their notable IC_50_ values. Specifically, the IC_50_ values were determined to be 0.107 ± 0.07 μM for compound 3h and 0.027 ± 0.002 μM for compound 3i, respectively.

**Table 2:** IC_50_ (μM) values of the synthesized compounds

Compound	AChE	BChE
	IC_50_ (μM)	IC_50_ (μM)
3a	>10	>10
3b	>10	>10
3c	3.679 ± 0.109	>10
3d	>10	>10
3e	1.867 ± 0.678	>10
3f	4.746 ± 0.098	>10
3g	9.957 ± 0.208	>10
3h	**0.107 ± 0.07**	>10
3i	**0.027 ± 0.002**	>10
Donepezil	0.0201 ± 0.001	—

### Cytotoxicity assay

Both the activity of a compound and its lack of cytotoxicity are criteria that determine whether it can be a potential drug candidate. To this end, the cytotoxic potential of the compounds was investigated on healthy fibroblast cells. For compounds 3h and 3i, the cytotoxicity values are IC_50_ = 36.062 ± 0.968 and 19.755 ± 0.627 μM, respectively (Table 3).

**Table 3:** IC_50_ values (μM) of compounds 3h and 3i against NIH/3T3 cell lines

Compound	NIH/3T3
3h	36.062 ± 0.968
3i	19.755 ± 0.627
Doxorubicin	>1000

**Fig. 1:**
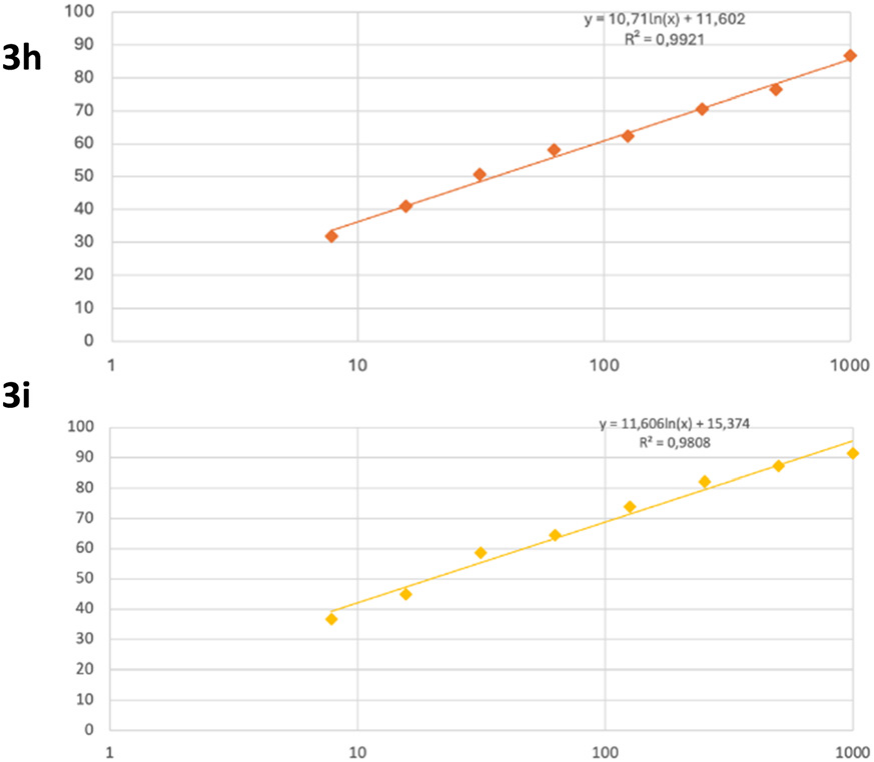
Cytotoxicity graphs of compounds 3h and 3i.

None of the compounds exhibited toxicity. The results obtained are from a 24 hour incubation period. The graphs of the IC_50_ values are presented in Fig. 1.

### Molecular docking

**Fig. 2:**
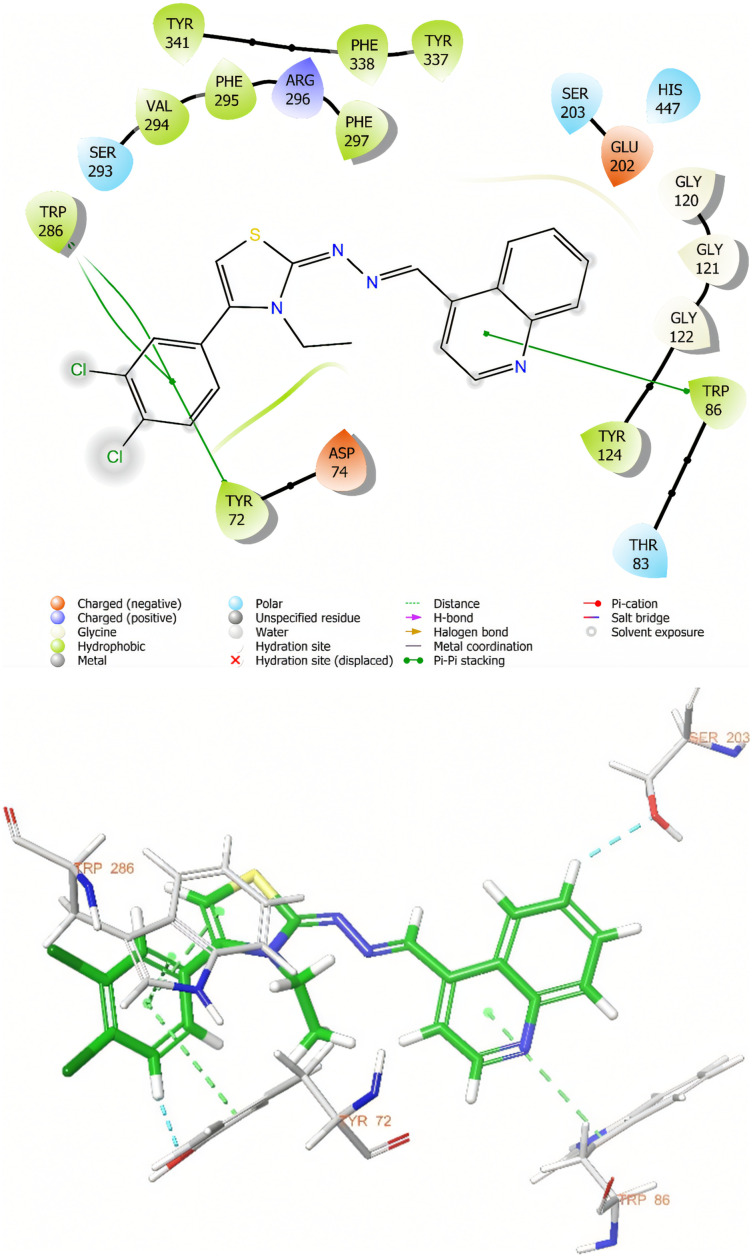
Two-dimensional and three-dimensional views of the interaction of compound 3h with the active site of the AChE enzyme.

**Fig. 3:**
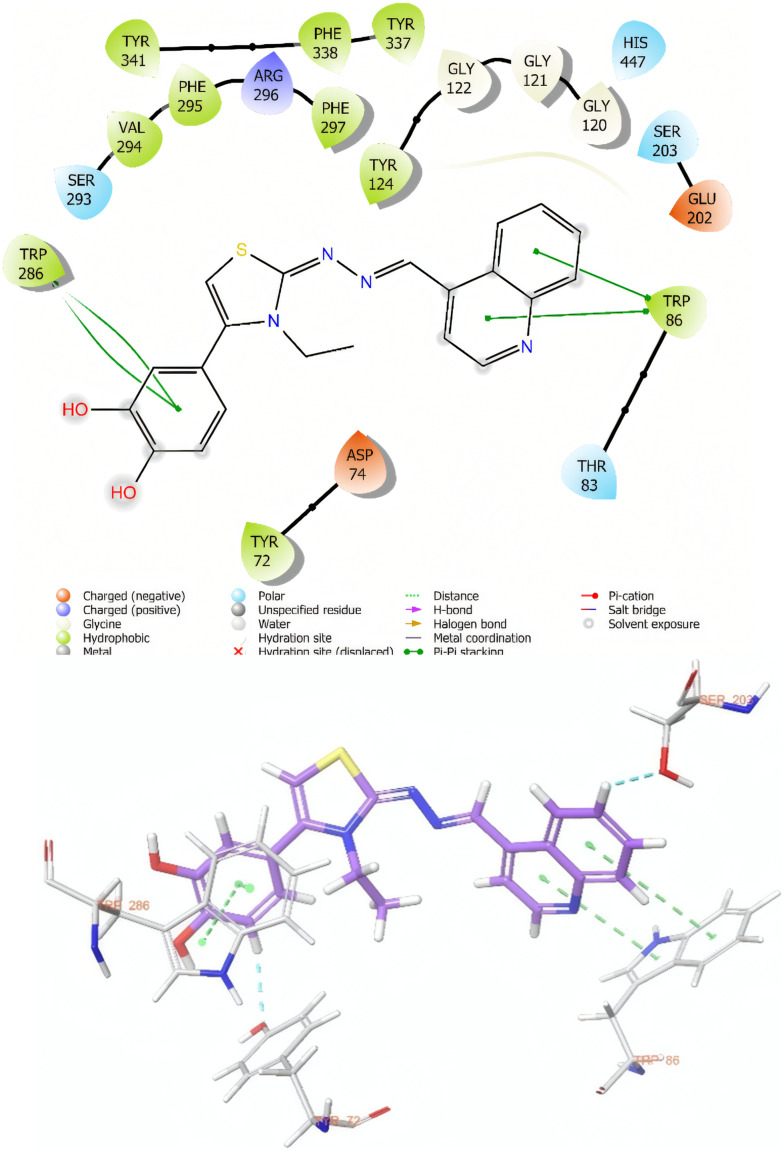
Two-dimensional and three-dimensional views of the interaction of compound 3i with the active site of the AChE enzyme.

As a result of molecular docking studies, compounds 3h and 3i have shown various interactions with the AChE enzyme. Both compounds interact with the TRP 86 amino acid in the cationic active site (CAS) of AChE through π–π interactions. The fact that donepezil also exhibits a similar interaction, and that compound 3i, which has the highest inhibitory potential among the compounds, interacts with this amino acid *via* two different points through its quinoxaline ring, highlights the significance of this interaction (Fig. 2 and 3).

A similar situation applies to the Trp286 amino acid located in the peripheral anionic site of AChE. Both compounds interact with the Trp286 amino acid, which is known to interact with donepezil, through π–π interactions.

### Molecular dynamics simulation

**Fig. 4:**
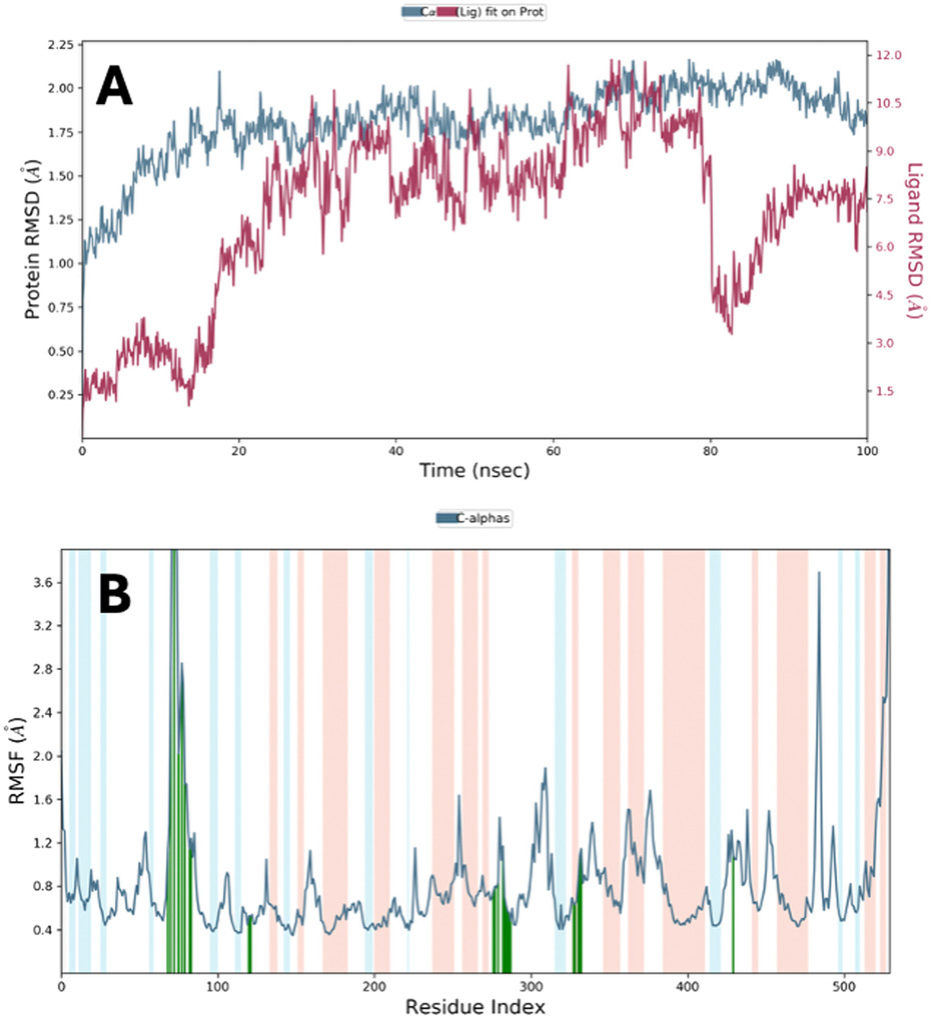
MD simulation results performed with the compound 3h–AChE complex (A and B).

**Fig. 5:**
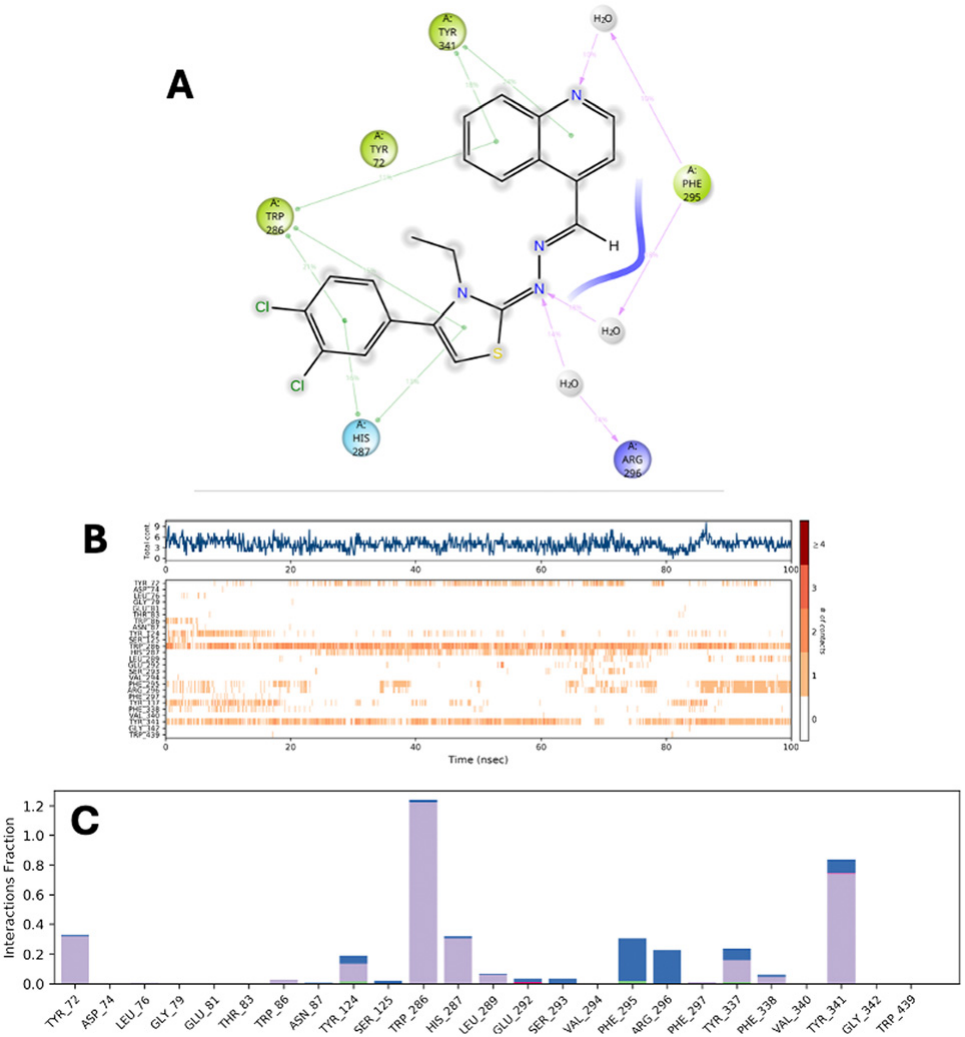
MD simulation results performed with the compound 3h–AChE complex (A–C).

**Fig. 6:**
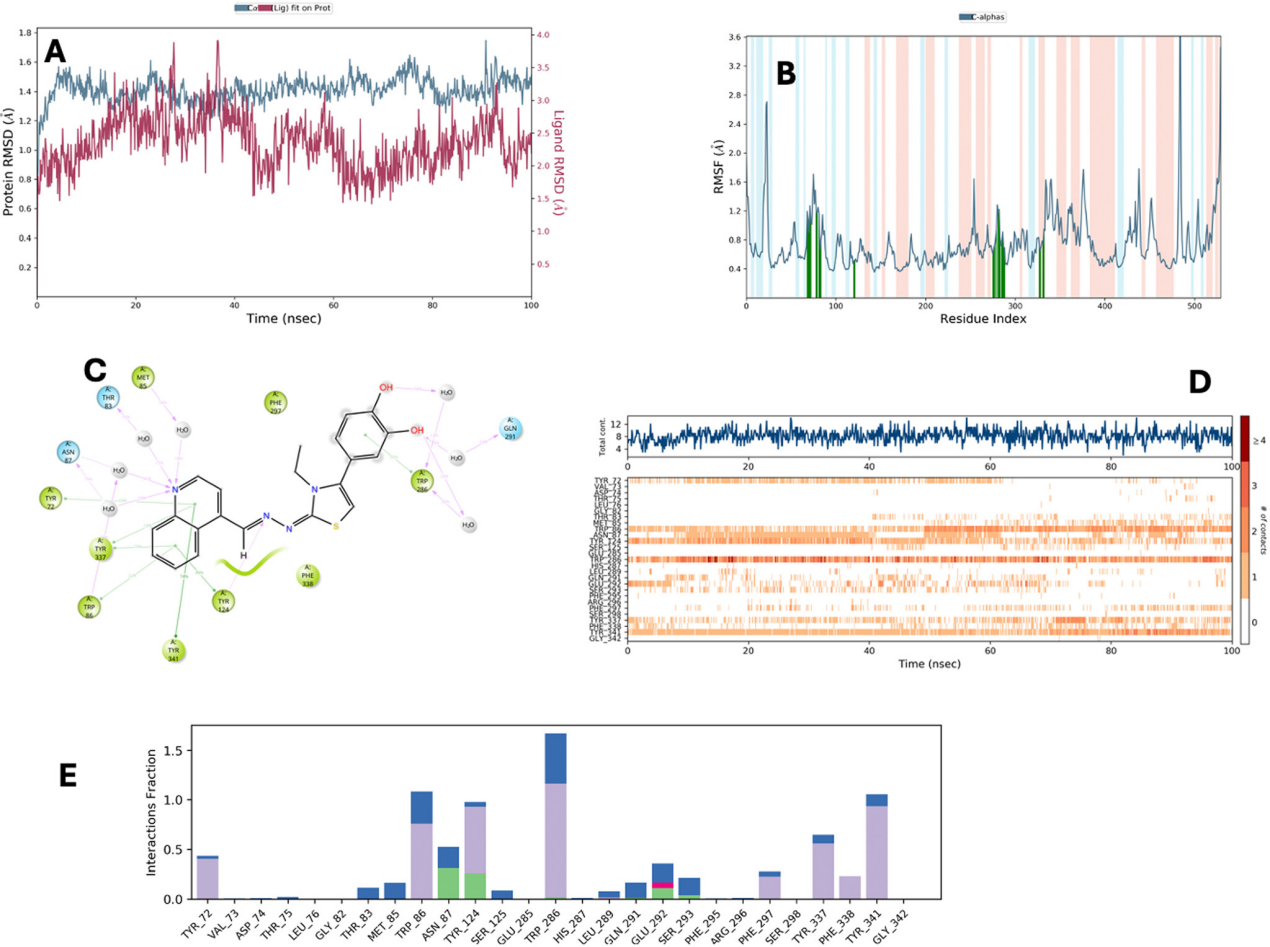
MD simulation results performed with the compound 3i–AChE complex (A–E).

Molecular dynamics (MD) simulations of 3h + 4EY7 and 3i + 4EY7 complexes were performed using the POPE membrane model for 100 ns. The obtained data are presented in Fig. 4–6. RMSD (root mean square deviation) analyses are in Fig. 4A and 6A, and RMSF (root mean square fluctuation) results are in Fig. 4B and 6B. Fig. 5A and 6C show the two-dimensional interaction analyses of the complexes, Fig. 5B and 6D show the time-dependent amino acid interactions, and Fig. 5C and 6E show the types of amino acid interactions.

The main difference between molecular dynamics simulations and docking studies is that they provide the opportunity to evaluate the continuity of interactions between the molecule and the target protein over time. This feature provides more detailed information about the stability of the systems. RMSD and RMSF parameters are important indicators in evaluating complex stability. An RMSD value of 3 Å or less indicates that the overall stability of the system is maintained. According to the data presented in Table 4, all complexes remain within this limit value. In the RMSF analysis, an increase can be observed in the loop regions, but being 1 Å or less in the α-helix and β-strand regions contributes to the preservation of structural integrity and the stability of the system. The relevant RMSF values are presented in Fig. 4B and 6B.

**Table 4:** RMSD and RMSF parameters and aromatic hydrogen bonds for compounds 3h and 3i

Comp.	RMSD	RMSF
3h	2.25 Å	Tyr72 (1.12 Å), Asp74 (2.83 Å), Leu76 (6.41 Å), Gly79 (2.02 Å), Glu81 (2.85 Å), Thr83 (1.74 Å), Trp86 (1.14 Å), Phe123 (0.45 Å), Gly126 (0.54 Å), Trp286 (0.75 Å), His287 (0.80 Å), Leu289 (0.83 Å), Glu292 (1.17 Å), Ser293 (0.71 Å), Val294 (0.63 Å), Phe295 (0.57 Å), Arg296 (0.55 Å), Phe297 (0.48 Å), Tyr337 (0.64 Å), Phe338 (0.63 Å), Tyr341 (1.09 Å), Gly342 (1.15 Å), Trp439 (1.08 Å)
3i	1.8 Å	Val73 (1.08 Å), Asp74 (0.90 Å), Thr75 (1.27 Å), Glu81 (1.49 Å), Thr83 (1.16 Å), Met85 (1.16 Å), Trp86 (0.67 Å), Asn87 (0.77 Å), Tyr124 (0.51 Å), Ser125 (0.52 Å), Glu285 (0.60 Å), Trp286 (0.69 Å), His287 (0.73 Å), Leu289 (0.94 Å), Gln291 (1.18 Å), Glu292 (1.22 Å), Ser293 (0.83 Å), Phe295 (0.80 Å), Arg296 (0.91 Å), Phe297 (0.70 Å), Ser298 (0.69 Å), Tyr337 (0.71 Å), Phe338 (0.66 Å), Tyr341 (0.74 Å), Gly342 (0.95 Å)

According to the data in Table 4 and Fig. 4B, the 3h compound exceeds the limits in terms of RMSF values in some regions, which leads to the questioning of the long-term stability of the relevant compound.

In order to be effective in AChE enzyme inhibition, the compound must bind strongly and continuously to both the catalytically active site (CAS) and the peripheral anionic site (PAS). In this context, amino acids Trp286 for PAS and Trp86 for CAS are of critical importance. The analyses show that compound 3h established sporadic interactions with these critical sites. However, compound 3i showed continuous and strong interactions with Trp86 and Trp286 amino acids for 100 ns. This supports the continuity of the inhibitory activity of 3i and ensures that it is fully localized to the active site of the enzyme. Therefore, compound 3i stands out as a potential AChE inhibitor candidate.

## Conclusions

In this study, novel and structurally unique thiazole/quinoline-derived compounds were successfully synthesized. The cholinesterase inhibition assays revealed that the synthesized compounds exhibited selective inhibitory activity toward acetylcholinesterase (AChE). Among them, compounds 3h and 3i demonstrated significant inhibitory potency, as indicated by their IC_50_ values.

In addition to their enzyme inhibitory activity, these two compounds were subjected to cytotoxicity evaluation and were classified as non-cytotoxic, supporting their potential safety profiles. Molecular docking studies were conducted to explore the binding characteristics of the compounds with AChE. It was observed that both compounds engaged in π–π stacking interactions between their quinoline moieties and the catalytically active site (CAS) of AChE, suggesting a key role in binding affinity.

Notably, molecular dynamics simulations indicated that compound 3i exhibited a more continuous and stable interaction profile compared to the other compound. This enhanced stability provides a plausible explanation for the compound's strong inhibitory activity (IC_50_ = 0.027 ± 0.002 μM), which is comparable to the reference drug donepezil (IC_50_ = 0.021 ± 0.001 μM).

Taken together, these findings highlight compound 3i as a promising lead candidate for further development as a selective and potent AChE inhibitor, with potential therapeutic relevance in the treatment of neurodegenerative disorders such as Alzheimer's disease.

## Experimental

### Chemistry

*N*-Ethylhydrazinecarbothioamide (1) was obtained by reacting excess ethyl isothiocyanate with hydrazine hydrate. After the obtained *N*-ethylhydrazinecarbothioamide (1) was crystallized from ethanol, it was refluxed with quinoline-4-carbaldehyde in absolute ethanol. The reaction, monitored by thin-layer chromatography (TLC), was completed by filtering the precipitated product. The product was then crystallized from ethanol, yielding *N*-ethyl-2-(quinolin-4-ylmethylene)hydrazine-1-carbothioamide (2).

In the third and final step, *N*-ethyl-2-(quinolin-4-ylmethylene)hydrazine-1-carbothioamide (2) was refluxed in absolute ethanol with appropriate phenacyl bromide derivatives. The reaction was monitored by TLC, and the precipitated products were separated by filtration and crystallized from ethanol. In this way, as indicated in Scheme 1, nine different quinoline/thiazole derivative compounds were obtained.

#### Synthesis of *N*-ethylhydrazinecarbothioamide (1)

Hydrazine hydrate (0.009 mol) was combined with ethyl isothiocyanate (0.003 mol, 0.2430 g) in absolute ethanol and heated under reflux at 80 °C for 4 hours. Upon completion of the reaction, the resulting solid was collected by filtration and rinsed with ethanol.

#### Synthesis of *N*-ethyl-2-(quinolin-4-ylmethylene)hydrazine-1-carbothioamide (2)

Quinoline-4-carbaldehyde (0.002 mol, 0.3141 g) was dissolved in absolute ethanol, followed by the addition of *N*-ethylhydrazinecarbothioamide (1) (0.002 mol, 0.2381 g). The resulting reaction mixture was heated under reflux for 12 hours. The progress of the reaction was monitored using TLC, and the product precipitated as a solid within the reaction medium. The solid was collected by filtration and washed with cold ethanol before being dried.

### Synthesis of the target compounds

*N*-Ethyl-2-(quinolin-4-ylmethylene)hydrazine-1-carbothioamide (2) (0.001 mol, 0.258 g) was dissolved in absolute ethanol, and then phenacyl bromide derivatives (0.001 mol) were added. The mixture was refluxed for 12 hours, during which the reaction progress was monitored *via* TLC. A solid product formed in the reaction medium, which was subsequently isolated by filtration, washed with cold ethanol, and dried.

#### 3-Ethyl-4-phenyl-2-((quinolin-4-ylmethylene)hydrazineylidene)-2,3-dihydrothiazole (3a)

Yield: 77%, M.p.: 146.2–146.6 °C. ^1^H-NMR (300 MHz, DMSO-*d*_6_): *δ* = 1.18 (3H, t, *J* = 7.06 Hz, –CH_3_), 3.97 (2H, q, *J* = 7.01 Hz, –CH_2_), 6.78 (1H, s, thiazole), 7.57 (5H, b.s., Ar–H), 7.87–7.93 (1H, m, Ar–H), 8.05–8.10 (1H, m, Ar–H), 8.18–8.22 (2H, m, Ar–H), 9.05–9.12 (3H, m, Ar–H). ^13^C-NMR (75 MHz, DMSO-*d*_6_): *δ* = 14.0, 41.8, 104.2, 118.4, 123.7, 125.3, 126.4, 129.4, 129.7, 130.2, 130.5, 133.5, 141.4, 144.2, 145.4, 174.1. HRMS (*m*/*z*): [M + H]^+^ calcd. for C_21_H_18_N_4_S: 359.1325; found: 359.1318.

#### 3-Ethyl-2-((quinolin-4-ylmethylene)hydrazineylidene)-4-(*p*-tolyl)-2,3-dihydrothiazole (3b)

Yield: 75%, M.p.: 137.6–138.1 °C. ^1^H-NMR (300 MHz, DMSO-*d*_6_): *δ* = 1.15 (3H, t, *J* = 7.03 Hz, –CH_3_), 2.39 (3H, s, phenyl-CH_3_), 3.88 (2H, q, *J* = 7.00 Hz, –CH_2_), 6.49 (1H, s, thiazole), 7.34 (2H, d, *J* = 8.04 Hz, 1,4-disubstituebenzene), 7.41 (2H, d, *J* = 8.16 Hz, 1,4-disubstituebenzene), 7.66–7.72 (1H, m, Ar–H), 7.78–7.82 (1H, m, Ar–H), 7.83–7.85 (1H, m, Ar–H), 8.08 (1H, dd, *J*_1_ = 0.99 Hz, *J*_2_ = 8.44 Hz, Ar–H), 8.92 (1H, s, –CH), 8.95 (1H, d, *J* = 4.53 Hz, Ar–H), 8.99 (1H, dd, *J*_1_ = 0.93 Hz, *J*_2_ = 8.53 Hz, Ar–H). ^13^C-NMR (75 MHz, DMSO-*d*_6_): *δ* = 13.8, 21.4, 41.2, 101.6, 120.4, 125.2, 125.8, 127.7, 128.1, 129.4, 129.9, 130.1, 138.8, 139.6, 140.9, 147.7, 149.0, 150.7, 171.9. HRMS (*m*/*z*): [M + H]^+^ calcd. for C_22_H_20_N_4_S: 373.1481; found: 373.1477.

#### 3-Ethyl-4-(4-methoxyphenyl)-2-((quinolin-4-ylmethylene)hydrazineylidene)-2,3-dihydrothiazole (3c)

Yield: 76%, M.p.: 151.1–151.5 °C. ^1^H-NMR (300 MHz, DMSO-*d*_6_): *δ* = 1.16 (3H, t, *J* = 6.97 Hz, –CH_3_), 3.83 (3H, s, –OCH_3_), 3.86–3.90 (2H, m, –CH_2_), 6.46 (1H, s, thiazole), 7.08 (2H, d, *J* = 8.58 Hz, 1,4-disubstituebenzene), 7.45 (2H, d, *J* = 8.58 Hz, 1,4-disubstituebenzene), 7.67–7.72 (1H, m, Ar–H), 7.79–7.82 (1H, m, Ar–H), 7.83–7.85 (1H, m, Ar–H), 8.08 (1H, dd, *J* = 8.35 Hz, Ar–H), 8.92 (1H, s, –NH), 8.94–9.00 (2H, m, Ar–H). ^13^C-NMR (75 MHz, DMSO-*d*_6_): *δ* = 13.8, 41.1, 55.7, 101.3, 114.7, 120.4, 123.0, 125.2, 125.8, 127.7, 129.9, 130.1, 131.0, 138.8, 140.7, 147.6, 149.0, 150.7, 160.4, 171.8. HRMS (*m*/*z*): [M + H]^+^ calcd. for C_22_H_20_N_4_OS: 389.1431; found: 389.1434.

#### 3-Ethyl-4-(4-nitrophenyl)-2-((quinolin-4-ylmethylene)hydrazineylidene)-2,3-dihydrothiazole (3d)

Yield: 79%, M.p.: 177.1–177.6 °C. ^1^H-NMR (300 MHz, DMSO-*d*_6_): *δ* = 1.20 (3H, t, *J* = 7.05 Hz, –CH_3_), 4.00 (2H, q, *J* = 6.98 Hz, –CH_2_), 6.98 (1H, s, thiazole), 7.86–7.92 (3H, m, Ar–H), 8.03–8.07 (1H, m, Ar–H), 8.18–8.22 (2H, m, Ar–H), 8.39 (2H, d, *J* = 8.83 Hz, Ar–H), 9.05 (1H, d, *J* = 8.29 Hz, Ar–H), 9.08 (1H, s, –NH), 9.13 (1H, d, *J* = 5.52 Hz, Ar–H). ^13^C-NMR (75 MHz, DMSO-*d*_6_): *δ* = 14.0, 42.0, 106.5, 118.8, 124.7, 124.5, 125.4, 126.3, 129.3, 130.8, 133.2, 136.7, 139.3, 142.0, 145.2, 145.5, 145.9, 148.3, 173.9. HRMS (*m*/*z*): [M + H]^+^ calcd. for C_21_H_17_N_5_O_2_S: 404.1176; found: 404.1181.

#### 3-Ethyl-4-(4-fluorophenyl)-2-((quinolin-4-ylmethylene)hydrazineylidene)-2,3-dihydrothiazole (3e)

Yield: 78%, M.p.: 126.7–127.2 °C. ^1^H-NMR (300 MHz, DMSO-*d*_6_): *δ* = 1.15 (3H, t, *J* = 7.04 Hz, –CH_3_), 3.87 (2H, q, *J* = 6.98 Hz, –CH_2_), 6.57 (1H, s, Thiazole), 7.36–7.42 (2H, m, Ar–H), 7.58–7.63 (2H, m, Ar–H), 7.68–7.73 (1H, m, Ar–H), 7.80–7.83 (1H, m, Ar–H), 7.85–7.87 (1H, m, Ar–H), 8.07–8.10 (1H, m, Ar–H), 8.94 (1H, s, –NH), 8.96 (1H, d, *J* = 4.56 Hz, Ar–H), 8.97–9.00 (1H, m, Ar–H). ^13^C-NMR (75 MHz, DMSO-*d*_6_): *δ* = 13.8, 41.2, 102.4, 116.2, 116.5, 120.3, 125.2, 125.8, 127.4, 127.8, 129.8, 130.1, 131.9, 132.0, 139.2, 139.8, 147.7, 150.5, 164.7. HRMS (*m*/*z*): [M + H]^+^ calcd. for C_21_H_17_N_4_FS: 377.1231; found: 377.1235.

#### 4-(4-Chlorophenyl)-3-ethyl-2-((quinolin-4-ylmethylene)hydrazineylidene)-2,3-dihydrothiazole (3f)

Yield: 78%, M.p.: 101.7–102.3 °C. ^1^H-NMR (300 MHz, DMSO-*d*_6_): *δ* = 1.15 (3H, t, *J* = 7.04 Hz, –CH_3_), 3.88 (2H, q, *J* = 6.99 Hz, –CH_2_), 6.60 (1H, s, thiazole), 7.56–7.63 (4H, m, Ar–H), 7.66–7.72 (1H, m, Ar–H), 7.78–7.82 (1H, m, Ar–H), 7.83–7.85 (1H, m, Ar–H), 8.08 (1H, dd, *J*_1_ = 0.91 Hz, *J*_2_ = 8.41 Hz, Ar–H), 8.93 (1H, s, –NH), 8.95 (1H, d, *J* = 4.56 Hz, Ar–H), 8.98 (1H, dd, *J*_1_ = 0.86 Hz, *J*_2_ = 8.54 Hz, Ar–H). ^13^C-NMR (75 MHz, DMSO-*d*_6_): *δ* = 13.8, 41.3, 102.7, 120.5, 125.1, 125.7, 127.7, 129.4, 129.8, 129.9, 130.1, 131.6, 134.7, 138.7, 139.6, 148.1, 149.0, 150.8, 171.8. HRMS (*m*/*z*): [M + H]^+^ calcd. for C_21_H_17_N_4_SCl: 393.0935; found: 393.0938.

#### 4-(3-Ethyl-2-((quinolin-4-ylmethylene)hydrazineylidene)-2,3-dihydrothiazol-4-yl)benzonitrile (3g)

Yield: 80%, M.p.: 193.3–194.1 °C. ^1^H-NMR (300 MHz, DMSO-*d*_6_): *δ* = 1.16 (3H, t, *J* = 7.04 Hz, –CH_3_), 3.91 (2H, q, *J* = 7.00 Hz, –CH_2_), 6.75 (1H, s, thiazole), 7.69–7.75 (1H, m, Ar–H), 7.76–7.78 (2H, m, Ar–H), 7.81–7.87 (1H, m, Ar–H), 7.89 (1H, d, *J* = 4.65 Hz, Ar–H), 8.03 (1H, d, *J* = 8.41 Hz, Ar–H), 8.09 (1H, dd, *J*_1_ = 0.93 Hz, *J*_2_ = 8.44 Hz, Ar–H), 8.96–9.00 (3H, m, Ar–H). ^13^C-NMR (75 MHz, DMSO-*d*_6_): *δ* = 13.8, 41.5, 104.4, 112.5, 118.9, 120.3, 125.2, 125.8, 127.9, 129.5, 130.3, 133.3, 135.4, 139.3, 148.0, 150.3, 172.0. HRMS (*m*/*z*): [M + H]^+^ calcd. for C_22_H_17_N_5_S: 384.1277; found: 384.1267.

#### 4-(3,4-Dichlorophenyl)-3-ethyl-2-((quinolin-4-ylmethylene)hydrazineylidene)-2,3-dihydrothiazole (3h)

Yield: 81%, M.p.: 254.7–255.6 °C. ^1^H-NMR (300 MHz, DMSO-*d*_6_): *δ* = 1.18 (3H, t, *J* = 7.07 Hz, –CH_3_), 3.99 (2H, q, *J* = 7.00 Hz, –CH_2_), 6.95 (1H, s, thiazole), 7.60 (2H, dd, *J*_1_ = 2.07 Hz, *J*_2_ = 8.32 Hz, Ar–H), 7.84 (1H, *J* = 8.31 Hz, Ar–H), 7.92–7.97 (2H, m, Ar–H), 8.11–8.16 (1H, m, Ar–H), 8.23–8.26 (1H, m, Ar–H), 8.29 (1H, d, *J* = 5.91 Hz, Ar–H), 9.08 (1H, d, *J* = 8.35 Hz, Ar–H), 9.11 (1H, s, –NH), 9.17 (1H, d, *J* = 5.84 Hz, Ar–H). ^13^C-NMR (75 MHz, DMSO-*d*_6_): *δ* = 14.0, 42.0106.2, 118.1, 122.3, 125.4, 126.5, 129.9, 130.0, 130.8, 131.6, 131.7, 132.2, 133.1, 134.3, 138.9, 139.7, 143.7, 144.2, 147.9, 174.4. HRMS (*m*/*z*): [M + H]^+^ calcd. for C_21_H_16_N_4_SCl_2_: 427.0545; found: 427.0509.

#### 4-(3-Ethyl-2-((quinolin-4-ylmethylene)hydrazineylidene)-2,3-dihydrothiazol-4-yl)benzene-1,2-diol (3i)

Yield: 74%, M.p.: 171.1–172.0 °C. ^1^H-NMR (300 MHz, DMSO-*d*_6_): *δ* = 7.75–7.80 (1H, m, Ar–H), 7.87–7.92 (1H, m, Ar–H), 8.08 (1H, d, *J* = 4.43 Hz, Ar–H), 8.15–8.18 (1H, m, Ar–H), 9.04 (1H, d, *J* = 8.33 Hz, Ar–H), 9.11 (1H, d, *J* = 4.40 Hz, Ar–H), 9.44 (1H, s, –CH). ^13^C-NMR (75 MHz, DMSO-*d*_6_): *δ* = 14.0, 14.9, 41.6, 102.2, 116.3, 116.8, 117.7, 118.9, 121.0, 121.3, 123.4, 125.3, 126.2, 128.4, 128.9, 131.1, 132.4, 136.6, 141.8, 144.8, 145.9, 146.7, 147.2, 149.1, 173.4, 177.3. HRMS (*m*/*z*): [M + H]^+^ calcd. for C_21_H_18_N_4_O_2_S: 391.1223; found: 391.1213.

### Anticholinesterase enzyme activity studies

The anticholinesterase enzyme activities of the compounds were investigated using the modified Ellman method. The details of this method have been presented in detail in our previous studies.^25–27^

### Cytotoxicity assay

To spectroscopically determine the effects of the synthesized compounds on cell viability, MTT analysis was performed. This method is based on the fact that metabolically active living cells reduce the colorless MTT (3-(4,5-dimethylthiazol-2-yl)-2,5-diphenyltetrazolium) salt, converting it into purple formazan crystals. In the analysis, healthy NIH3T3 cell lines were seeded into 96-well plates at a cell density of 1 × 10^6^ cells per well and incubated with the compounds for 24 hours.^28–33^

### Molecular docking

Among the obtained compounds, two compounds stood out in *in vitro* experiments: compound 3h and compound 3i. Therefore, molecular docking studies were conducted to examine the binding profiles of these two compounds to the AChE enzyme. The crystal structure PDB ID: 4EY7 (ref. 12) was used in the molecular docking studies. The molecular docking studies were initiated by preparing the protein structure using the Schrödinger Suite's “Protein Preparation Wizard” interface.^34^ Necessary optimization and minimization processes were performed using the OPLS3e force field, according to pH 7.4 ± 2. A suitable grid was created on the protein using the “Glide” interface. Compounds 3h and 3i were made suitable for molecular docking studies using “LigPrep”.^35^ Then, single precision (SP) docking studies were performed again using the “Glide” interface. The molecular docking results were examined using the “structure analysis” interface, and two-dimensional poses were obtained in this way.^36,37^

### Molecular dynamics simulation

In this study, molecular dynamics (MD) simulations were conducted to evaluate the time-dependent stability of compound 3g within the active site of its target receptor. As a widely used computational approach, MD simulations help validate docking results by assessing the dynamic behavior of ligand–receptor complexes. A 100 nanosecond simulation was performed using the Desmond simulation package.^38^

The system was solvated with a TIP3P three-point water model and parameterized using the OPLS3e force field from the Schrödinger Suite, which ensures accurate modeling of intermolecular interactions and energy minimization. To neutralize the system and simulate physiological conditions, Na^+^ and Cl^−^ ions were added, resulting in a final salt concentration of 0.15 M.^39^

Simulations were carried out under the NPT ensemble (constant number of particles, pressure, and temperature), with the temperature maintained at 310.55 K and the pressure at 1.01325 bar. The RESPA integrator was used to solve the equations of motion.^40^ Pressure control was achieved through the Martyna–Tuckerman–Klein (MTK) method,^41^ while the Nose–Hoover thermostat maintained thermal stability.^42^

Electrostatic interactions over long distances were computed using the particle mesh Ewald (PME) method,^43^ and a cutoff of 9.0 Å was set for van der Waals and short-range electrostatic interactions. Desmond's default relaxation protocol—which includes constrained minimizations followed by short MD runs—was applied to equilibrate the system.

Following system preparation, the production MD simulation was executed under the defined conditions. Post-simulation, the structural stability and flexibility of the protein–ligand complex were analyzed through key metrics such as root mean square deviation (RMSD), root mean square fluctuation (RMSF), and radius of gyration (*R*_g_).

## Author contributions

Berkant Kurban: conceptualization, methodology, data curation. Derya Osmaniye: conceptualization, methodology, software, validation, formal analysis, investigation, resources, data curation, writing – original draft preparation, visualization. Serkan Levent: methodology, formal analysis, writing – original draft preparation. Yusuf Özkay: resources, writing – review and editing, supervision. Zafer Asım Kaplancıklı: conceptualization, investigation, resources, writing – original draft preparation, visualization, supervision.

## Conflicts of interest

There are no conflicts to declare.

## Supplementary Material

MD-OLF-D6MD00165C-s001

## Data Availability

The data supporting this article have been included as part of the supplementary information (SI). Supplementary information: ^1^H-NMR, ^13^C-NMR, and HRMS spectra. See DOI: https://doi.org/10.1039/d6md00165c.
