# Supplementary material for: Quinoline/thiazole compounds as selective acetylcholinesterase inhibitors: synthesis and biological assessment
Source: RSC Med Chem. 2026 Apr 7;17(5):2404–12. doi: 10.1039/d6md00165c (PMC13107341; doi:10.1039/d6md00165c)
Supplement: MD-017-D6MD00165C-s001 [file MD-017-D6MD00165C-s001.pdf]

Supplementary Information (SI) for RSC Medicinal Chemistry.
This journal is © The Royal Society of Chemistry 2026

SUPPORTING INFORMATION

Quinoline/Thiazole Compounds as Selective Acetylcholinesterase Inhibitors: Synthesis and Biological Assessment

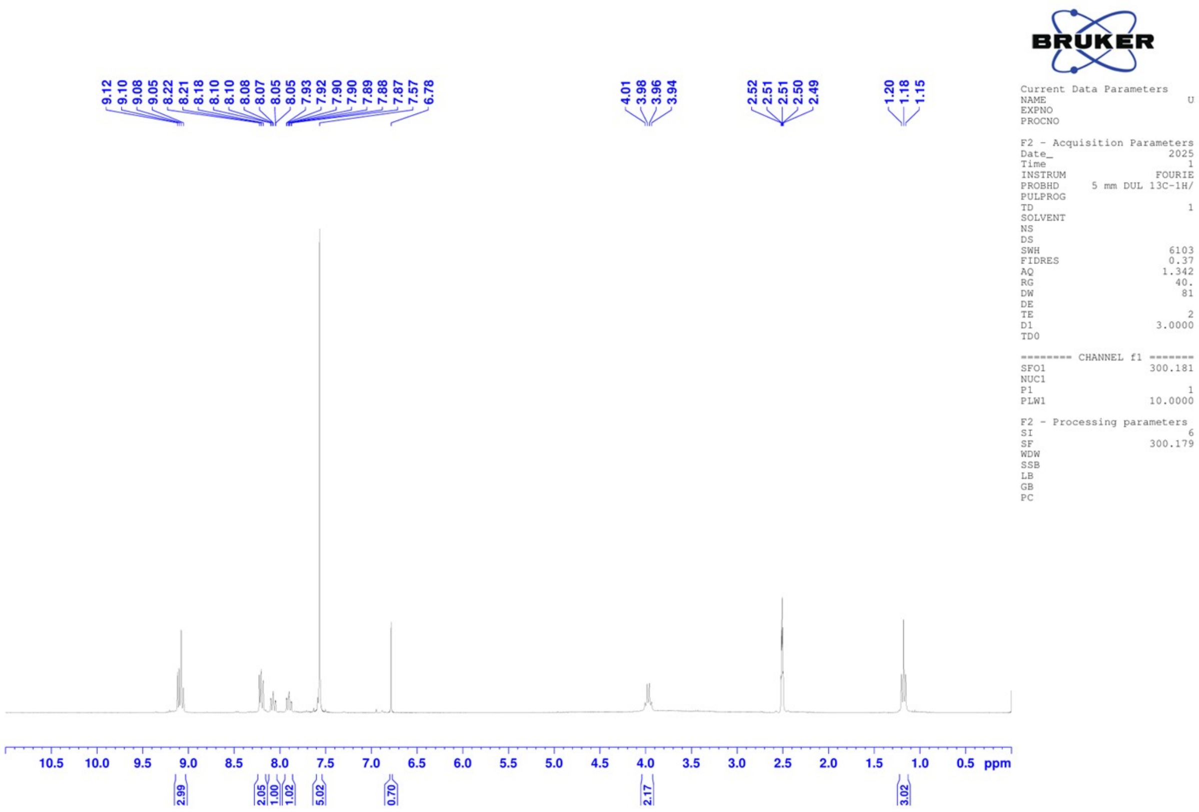

Spectra 1. ^{1}H-NMR spectra of compound **3a**

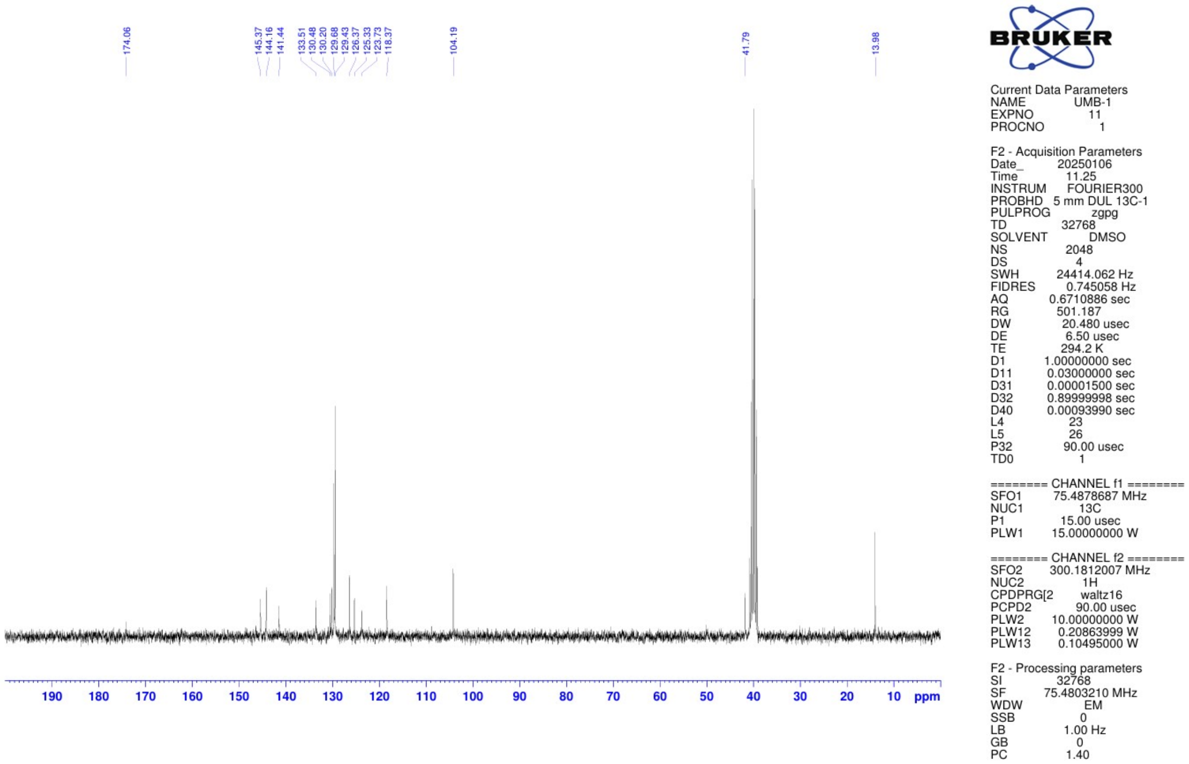

Spectra 2. ^{13}C-NMR spectra of compound **3a**

Data File: C:\LabSolutions\Data\Analiz\Derya\UMB-1_31.lcd

Elmt	Val.	Min	Max	Elmt	Val.	Min	Max	Elmt	Val.	Min	Max	Elmt	Val.	Min	Max	Use Adduct
H	1	5	35	O	2	0	0	S	2	0	4	Ru	2	0	0	H
C	4	9	35	F	1	0	0	Cl	1	0	0	Pd	2	0	0	
N	3	0	4	P	3	0	0	Br	1	0	0	I	3	0	0	

Error Margin (ppm):	5	DBE Range:	0.0 - 20.0	Electron Ions:	both
HC Ratio:	unlimited	Apply N Rule:	yes	Use MSn Info:	yes
Max Isotopes:	3	Isotope RI (%):	1.00	Isotope Res:	9000
MSn Iso RI (%):	10.00	MSn Logic Mode:	AND	Max Results:	50

Event#: 1 MS(E+) Ret. Time : 2.307 - 1.507 -> 2.110 Scan# : 347 - 227 -> 317

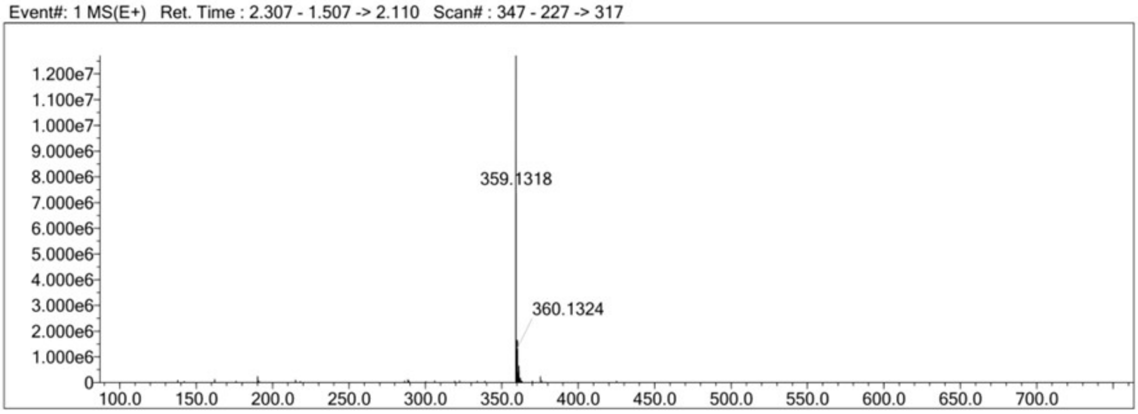

Measured region for 359.1318 m/z

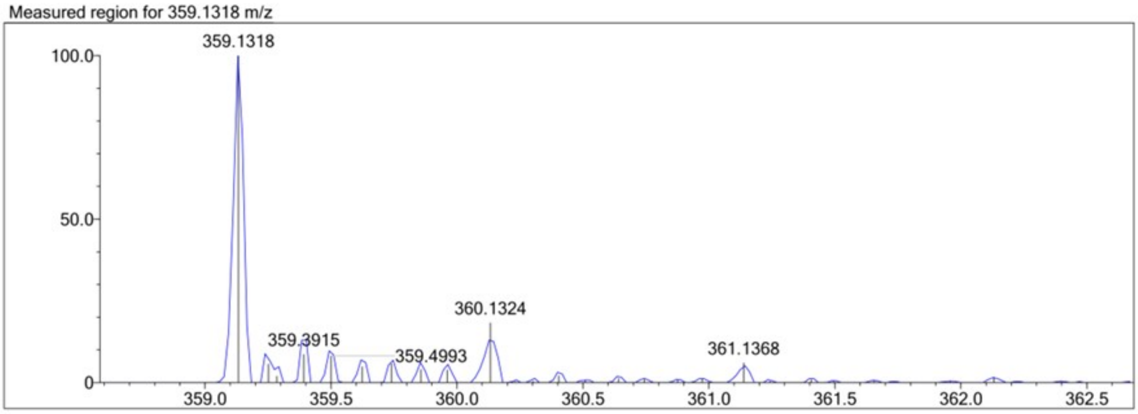

C21 H18 N4 S [M+H]+ : Predicted region for 359.1325 m/z

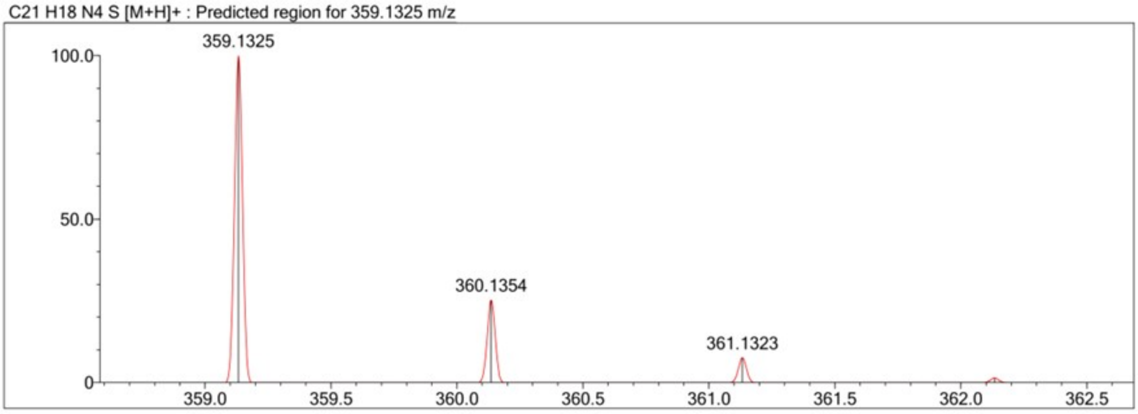

Rank	Score	Formula (M)	Ion	Meas. m/z	Pred. m/z	Df. (mDa)	Df. (ppm)	Iso	DBE
1	61.17	C21 H18 N4 S	[M+H]+	359.1318	359.1325	-0.7	-1.95	62.65	15.0

Spectra 3. HRMS spectra of compound **3a**

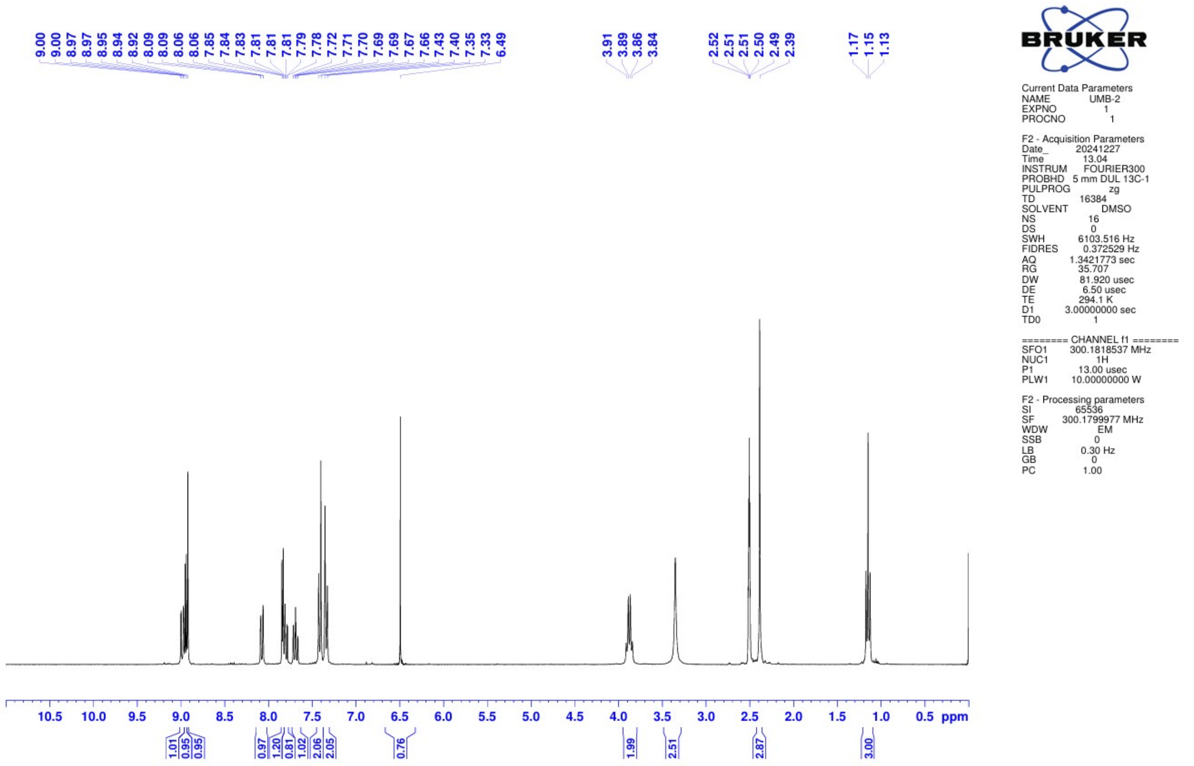

Spectra 4. ^{1}H-NMR spectra of compound **3b**

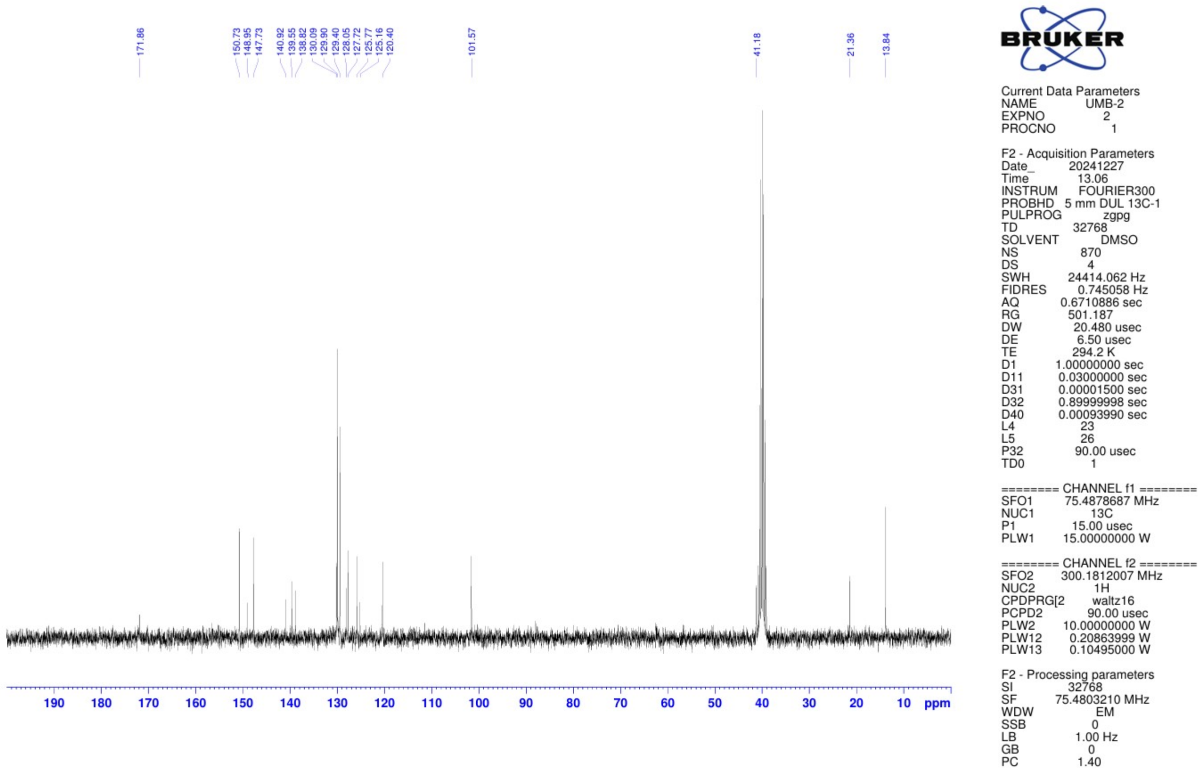

Spectra 5. ^{13}C-NMR spectra of compound **3b**

Data File: C:\LabSolutions\Data\Analiz\Derya\UMB-2_22.lcd

Elmt	Val.	Min	Max	Elmt	Val.	Min	Max	Elmt	Val.	Min	Max	Elmt	Val.	Min	Max	Use Adduct
H	1	5	35	O	2	0	4	S	2	0	2	Ru	2	0	0	H
C	4	9	35	F	1	0	0	Cl	1	0	0	Pd	2	0	0	
N	3	4	8	P	3	0	0	Br	1	0	0	I	3	0	0	

Error Margin (ppm):	5	DBE Range:	8.0 - 30.0	Electron Ions:	both
HC Ratio:	unlimited	Apply N Rule:	yes	Use MSn Info:	yes
Max Isotopes:	3	Isotope RI (%):	1.00	Isotope Res:	9000
MSn Iso RI (%):	10.00	MSn Logic Mode:	AND	Max Results:	50

Event#: 1 MS(E+) Ret. Time : 5.493 Scan# : 825

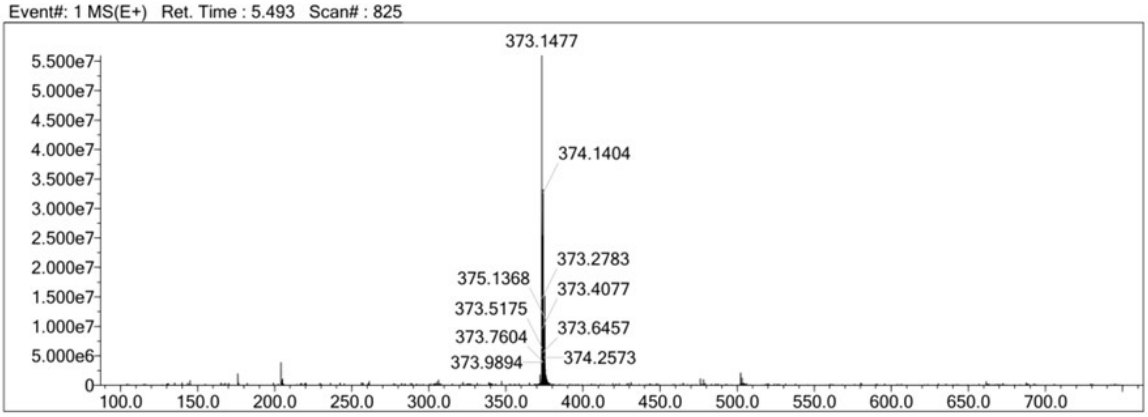

Measured region for 373.1477 m/z

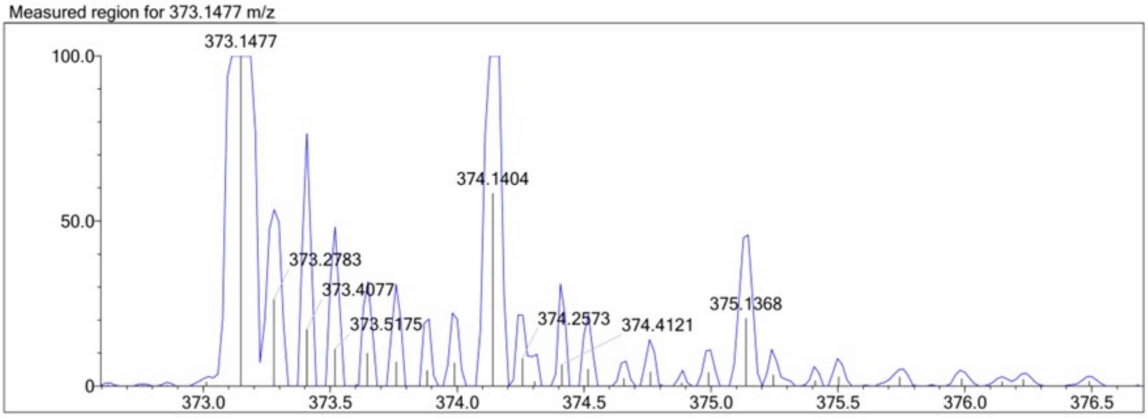

C22 H20 N4 S [M+H]+ : Predicted region for 373.1481 m/z

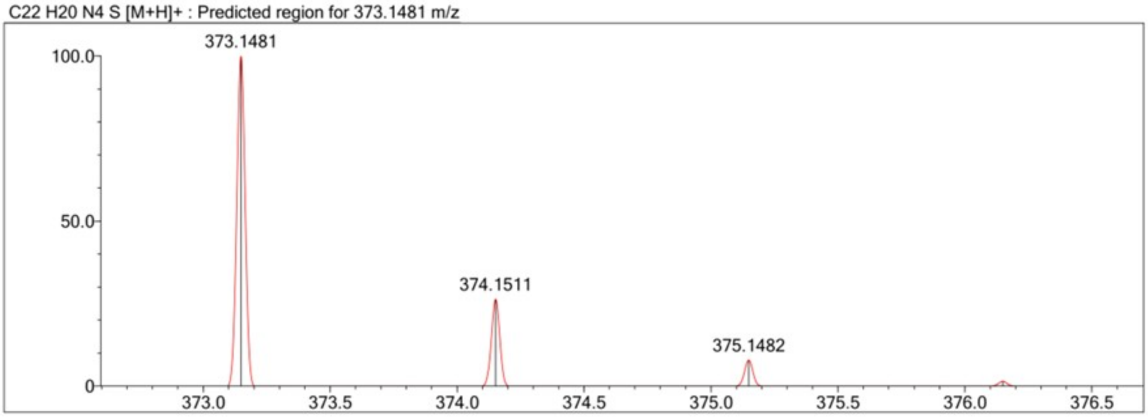

Rank	Score	Formula (M)	Ion	Meas. m/z	Pred. m/z	Df. (mDa)	Df. (ppm)	Iso	DBE
1	26.28	C22 H20 N4 S	[M+H]+	373.1477	373.1481	-0.4	-1.07	26.32	15.0

Spectra 6. HRMS spectra of compound **3b**

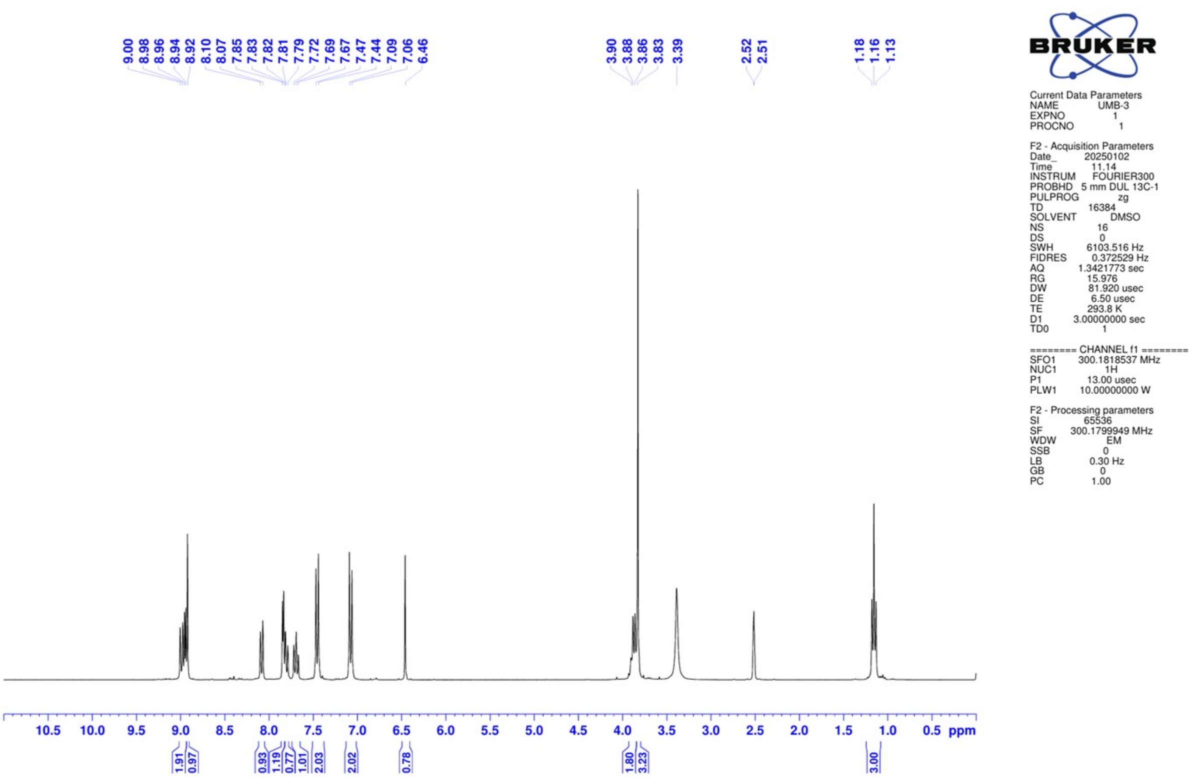

Spectra 7. ^{1}H-NMR spectra of compound **3c**

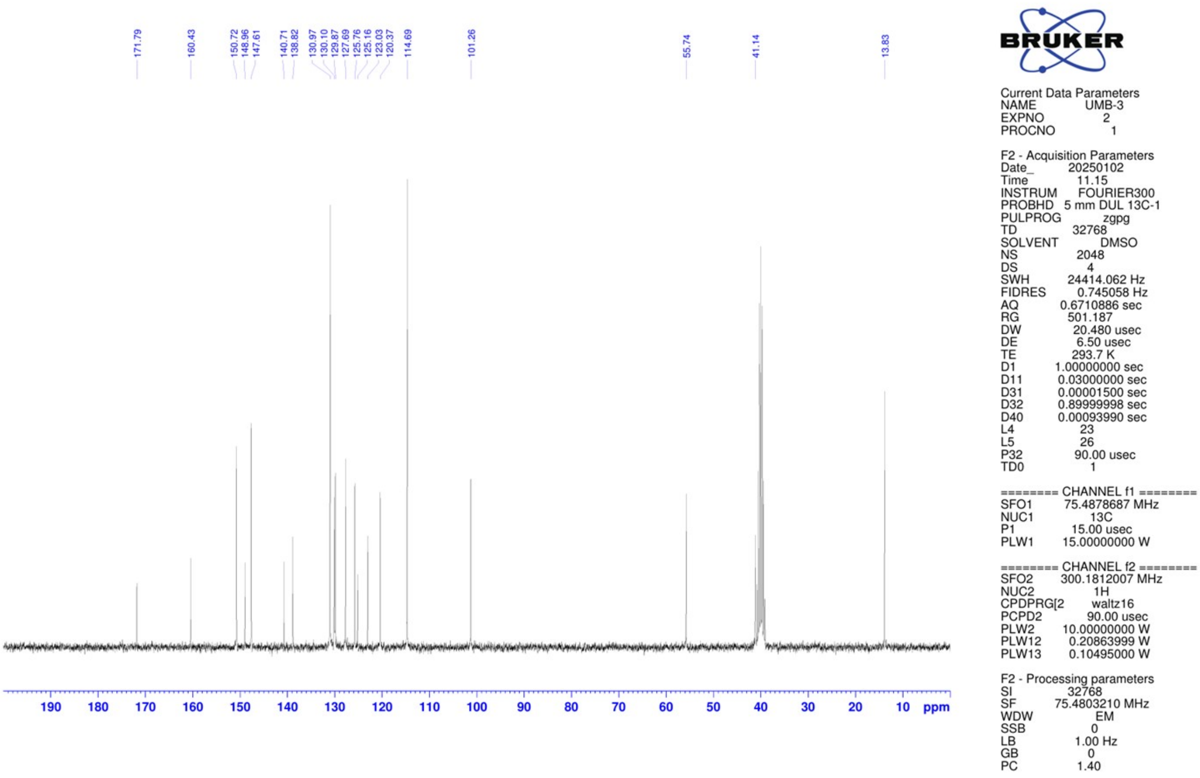

Spectra 8. ^{13}C-NMR spectra of compound **3c**

Formula Predictor Report - UMB-3_23.lcd Page 1 of 1

Data File: C:\LabSolutions\Data\Analiz\Derya\UMB-3_23.lcd

Elmt	Val.	Min	Max	Elmt	Val.	Min	Max	Elmt	Val.	Min	Max	Elmt	Val.	Min	Max	Use Adduct
H	1	5	35	O	2	0	4	S	2	0	2	Ru	2	0	0	H
C	4	9	35	F	1	0	0	Cl	1	0	0	Pd	2	0	0	
N	3	4	8	P	3	0	0	Br	1	0	0	I	3	0	0	

Error Margin (ppm): 5
HC Ratio: unlimited
Max Isotopes: 3
MSn Iso RI (%): 10.00

DBE Range: 8.0 - 30.0
Apply N Rule: yes
Isotope RI (%): 1.00
MSn Logic Mode: AND

Electron Ions: both
Use MSn Info: yes
Isotope Res: 9000
Max Results: 50

Event#: 1 MS(E+) Ret. Time : 4.173 Scan# : 627

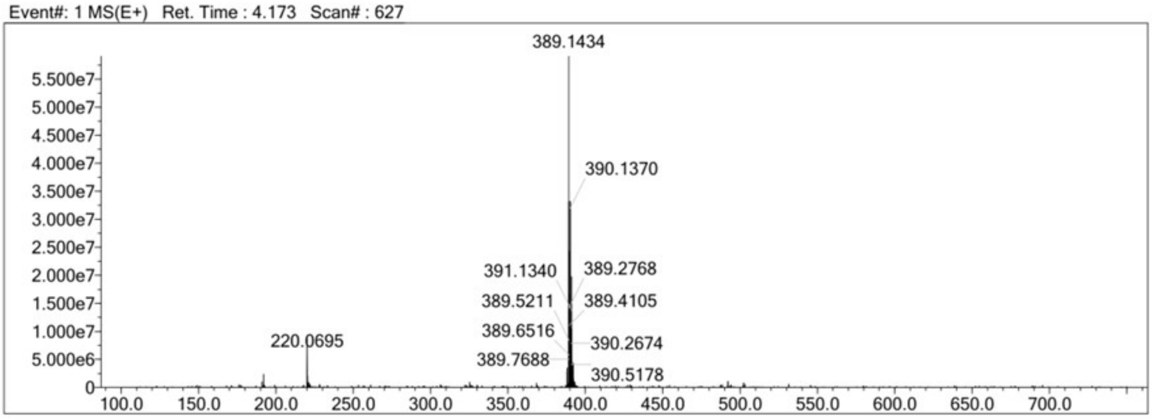

Measured region for 389.1434 m/z

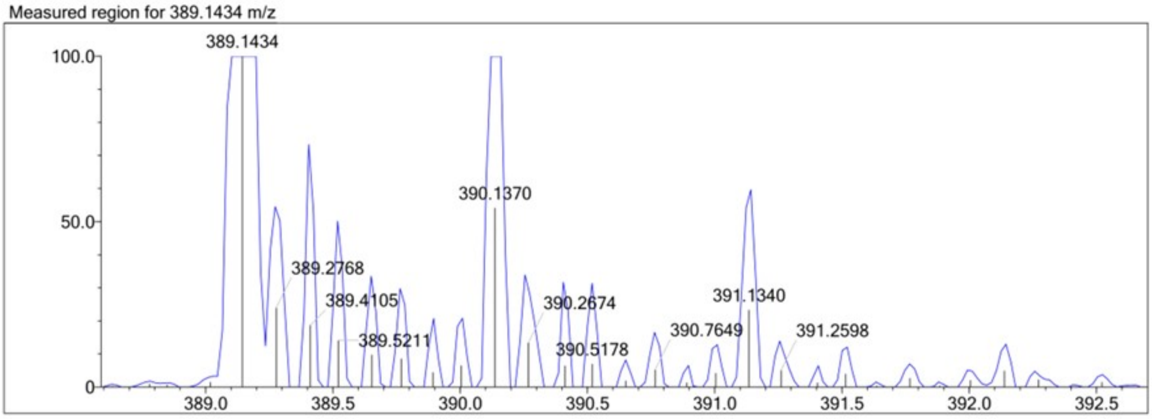

C22 H20 N4 O S [M+H]+ : Predicted region for 389.1431 m/z

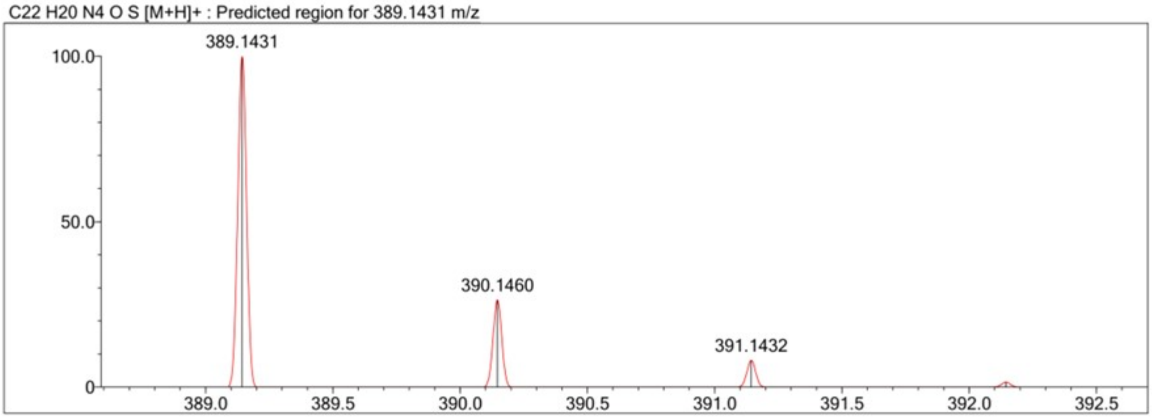

Rank	Score	Formula (M)	Ion	Meas. m/z	Pred. m/z	Df. (mDa)	Df. (ppm)	Iso	DBE
1	28.35	C22 H20 N4 O S	[M+H]+	389.1434	389.1431	0.3	0.77	28.35	15.0

Spectra 9. HRMS spectra of compound **3c**

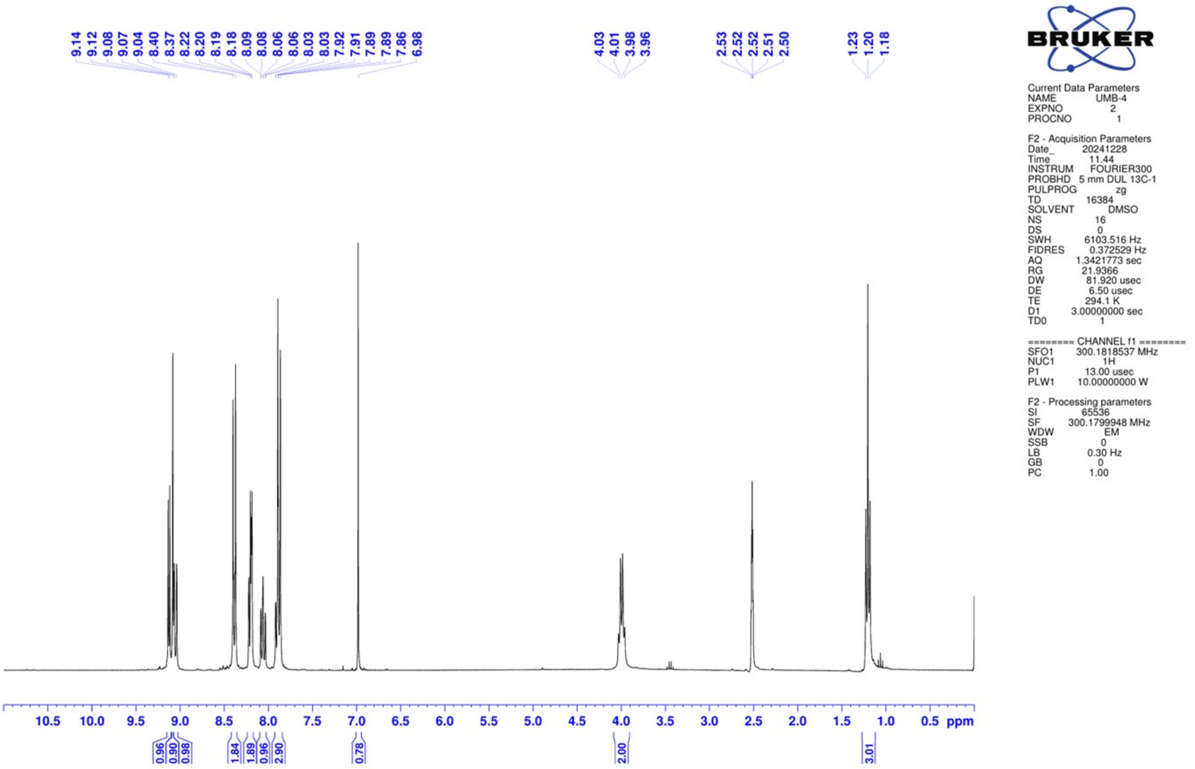

Spectra 10. ¹H-NMR spectra of compound **3d**

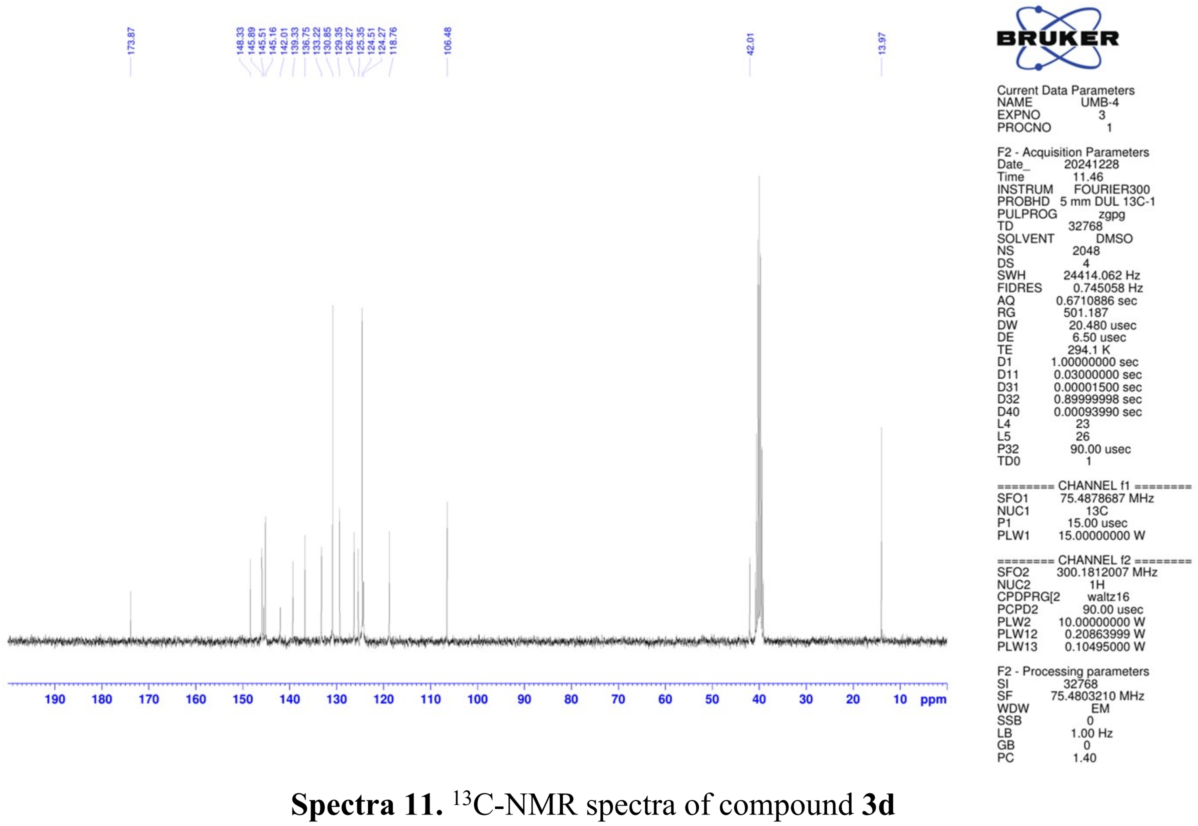

Spectra 11. ¹³C-NMR spectra of compound **3d**

Data File: C:\LabSolutions\Data\Analiz\Derya\UMB-4_24.lcd

Elmt	Val.	Min	Max	Elmt	Val.	Min	Max	Elmt	Val.	Min	Max	Elmt	Val.	Min	Max	Use Adduct
H	1	5	35	O	2	0	4	S	2	0	2	Ru	2	0	0	H
C	4	9	35	F	1	0	0	Cl	1	0	0	Pd	2	0	0	
N	3	4	8	P	3	0	0	Br	1	0	0	I	3	0	0	

Error Margin (ppm):	5	DBE Range:	8.0 - 30.0	Electron Ions:	both
HC Ratio:	unlimited	Apply N Rule:	yes	Use MSn Info:	yes
Max Isotopes:	3	Isotope RI (%):	1.00	Isotope Res:	9000
MSn Iso RI (%):	10.00	MSn Logic Mode:	AND	Max Results:	50

Event#: 1 MS(E+) Ret. Time : 4.280 Scan# : 643

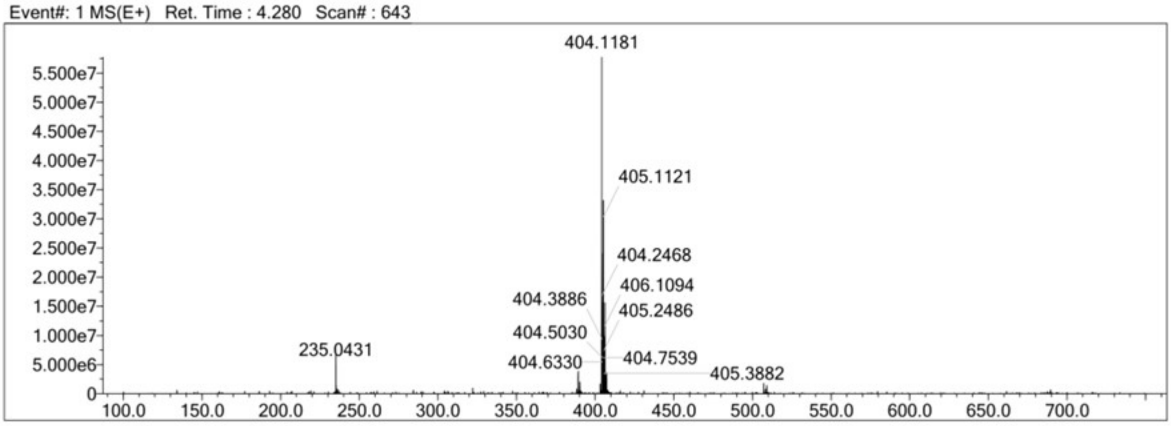

Measured region for 404.1181 m/z

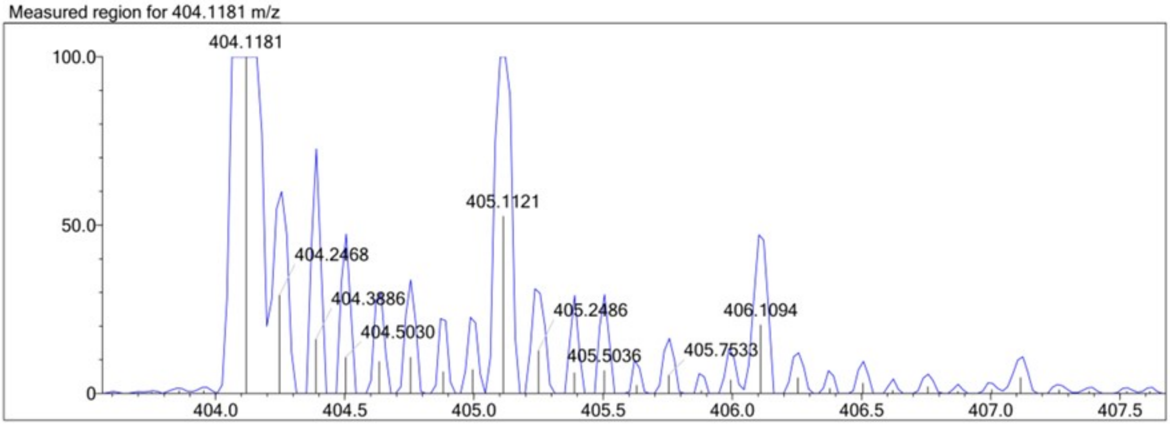

C21 H17 N5 O2 S [M+H]+ : Predicted region for 404.1176 m/z

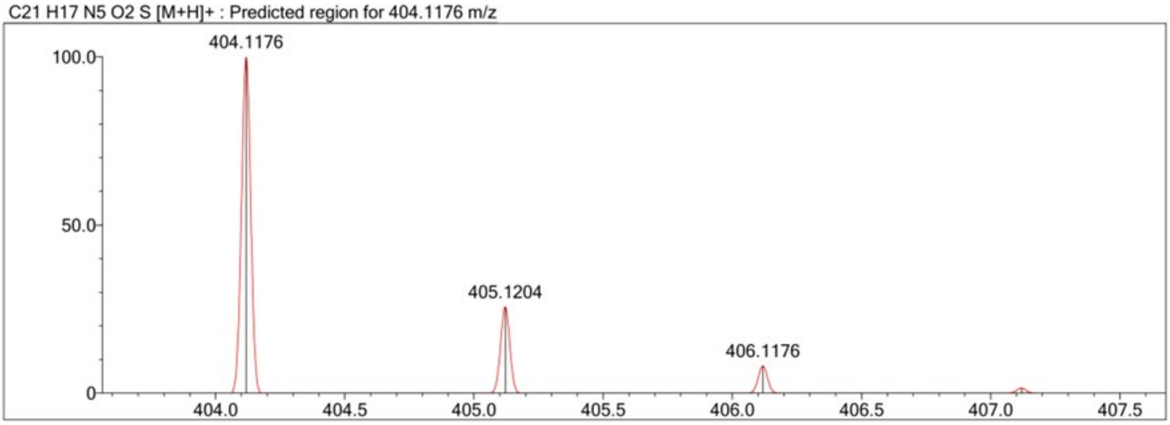

Rank	Score	Formula (M)	Ion	Meas. m/z	Pred. m/z	Df. (mDa)	Df. (ppm)	Iso	DBE
1	29.78	C21 H17 N5 O2 S	[M+H]+	404.1181	404.1176	0.5	1.24	29.96	16.0

Spectra 12. HRMS spectra of compound **3d**

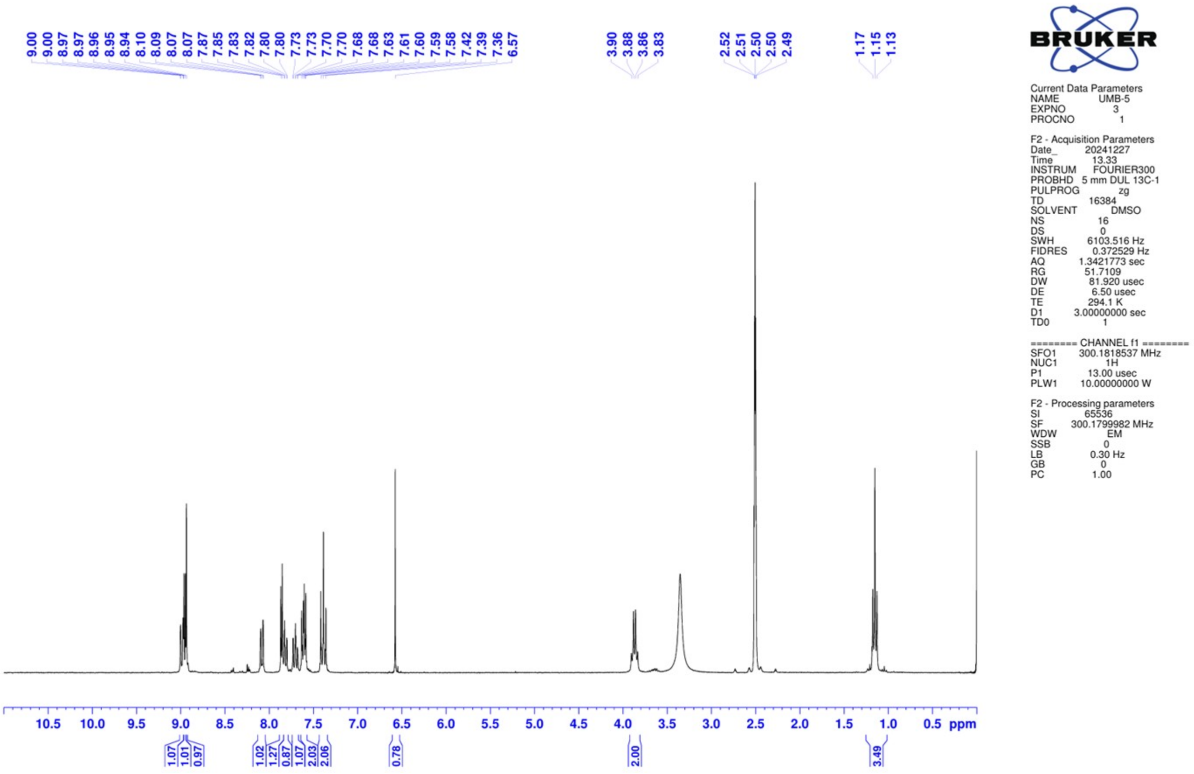

Spectra 13. ^{1}H-NMR spectra of compound **3e**

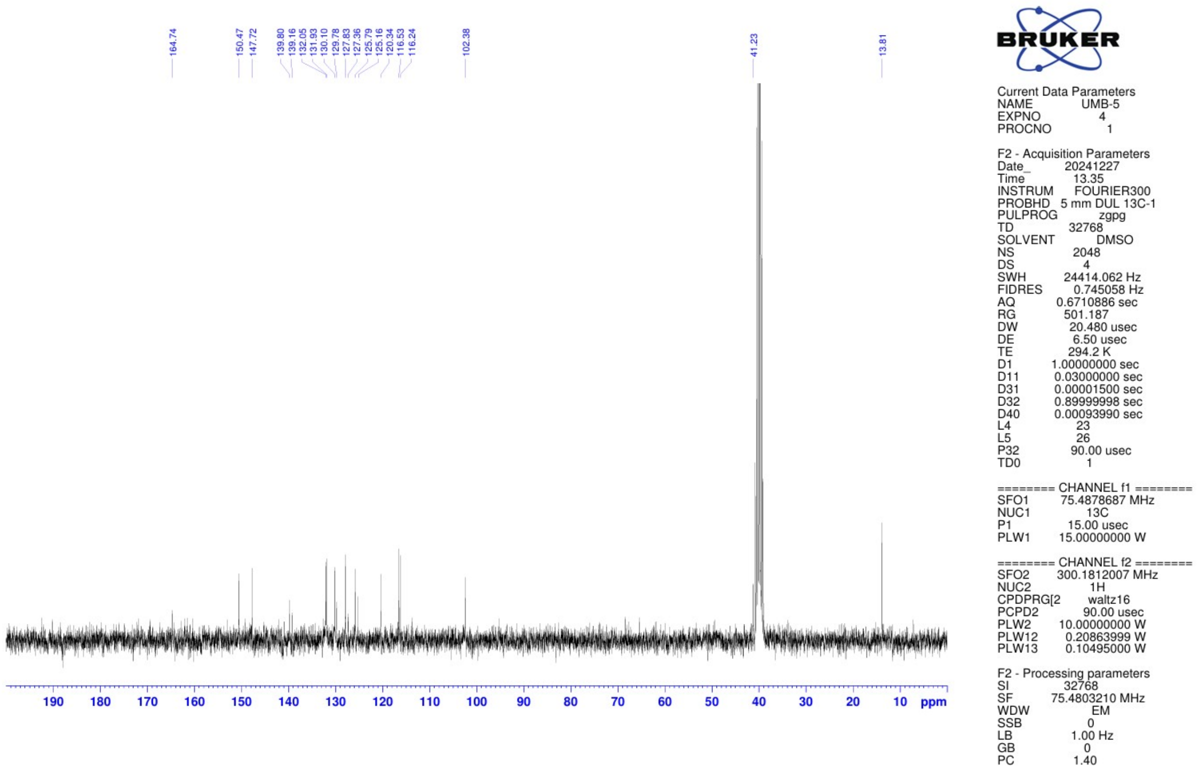

Spectra 14. ^{13}C-NMR spectra of compound **3e**

Data File: C:\LabSolutions\Data\Analiz\Derya\UMB-5_25.lcd

Elmt	Val.	Min	Max	Elmt	Val.	Min	Max	Elmt	Val.	Min	Max	Elmt	Val.	Min	Max	Use Adduct
H	1	5	35	O	2	0	3	S	2	0	2	Ru	2	0	0	H
C	4	9	35	F	1	0	1	Cl	1	0	0	Pd	2	0	0	
N	3	4	8	P	3	0	0	Br	1	0	0	I	3	0	0	

Error Margin (ppm):	5	DBE Range:	8.0 - 30.0	Electron Ions:	both
HC Ratio:	unlimited	Apply N Rule:	yes	Use MSn Info:	yes
Max Isotopes:	3	Isotope RI (%):	1.00	Isotope Res:	9000
MSn Iso RI (%):	10.00	MSn Logic Mode:	AND	Max Results:	50

Event#: 1 MS(E+) Ret. Time : 4.307 Scan# : 647

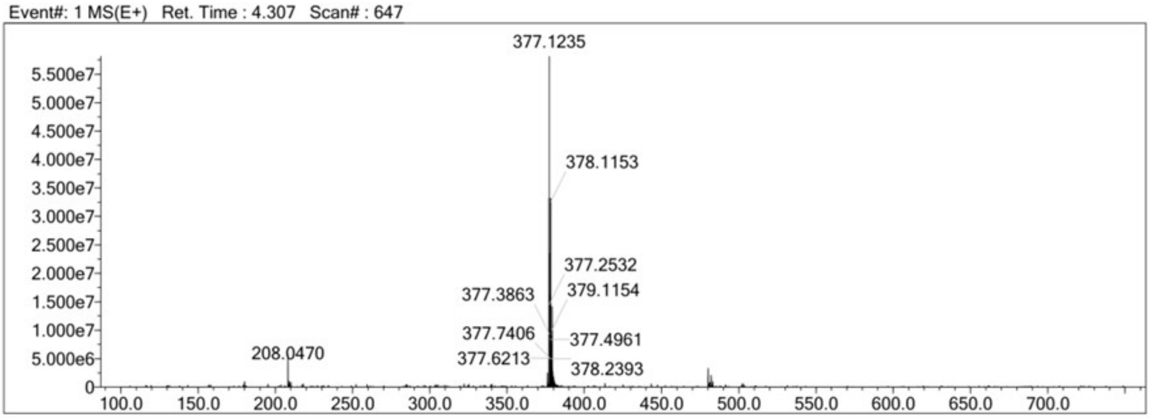

Measured region for 377.1235 m/z

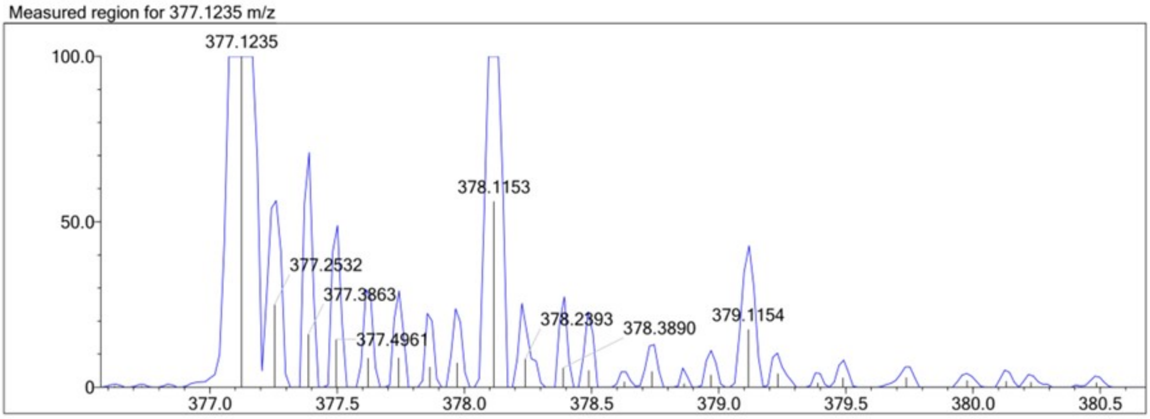

C21 H17 N4 F S [M+H]+ : Predicted region for 377.1231 m/z

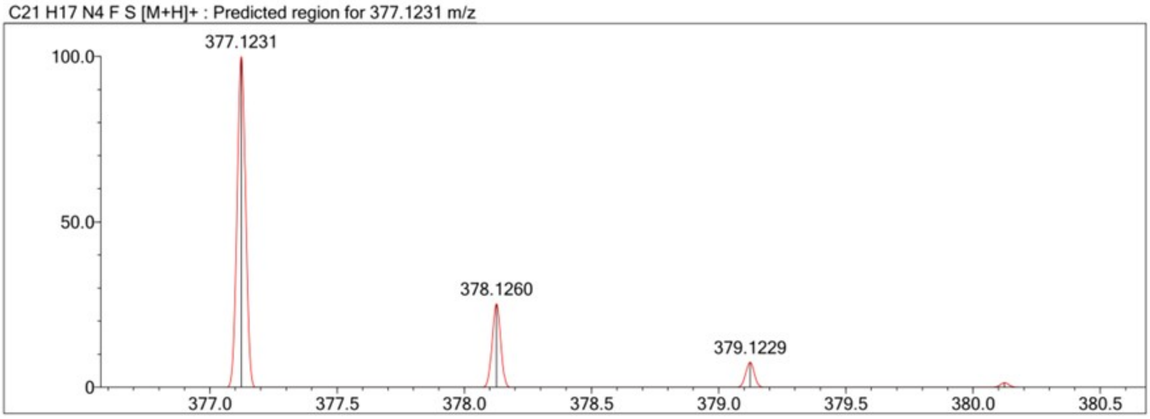

Rank	Score	Formula (M)	Ion	Meas. m/z	Pred. m/z	Df. (mDa)	Df. (ppm)	Iso	DBE
1	26.07	C21 H17 N4 F S	[M+H]+	377.1235	377.1231	0.4	1.06	26.11	15.0

Spectra 15. HRMS spectra of compound **3e**

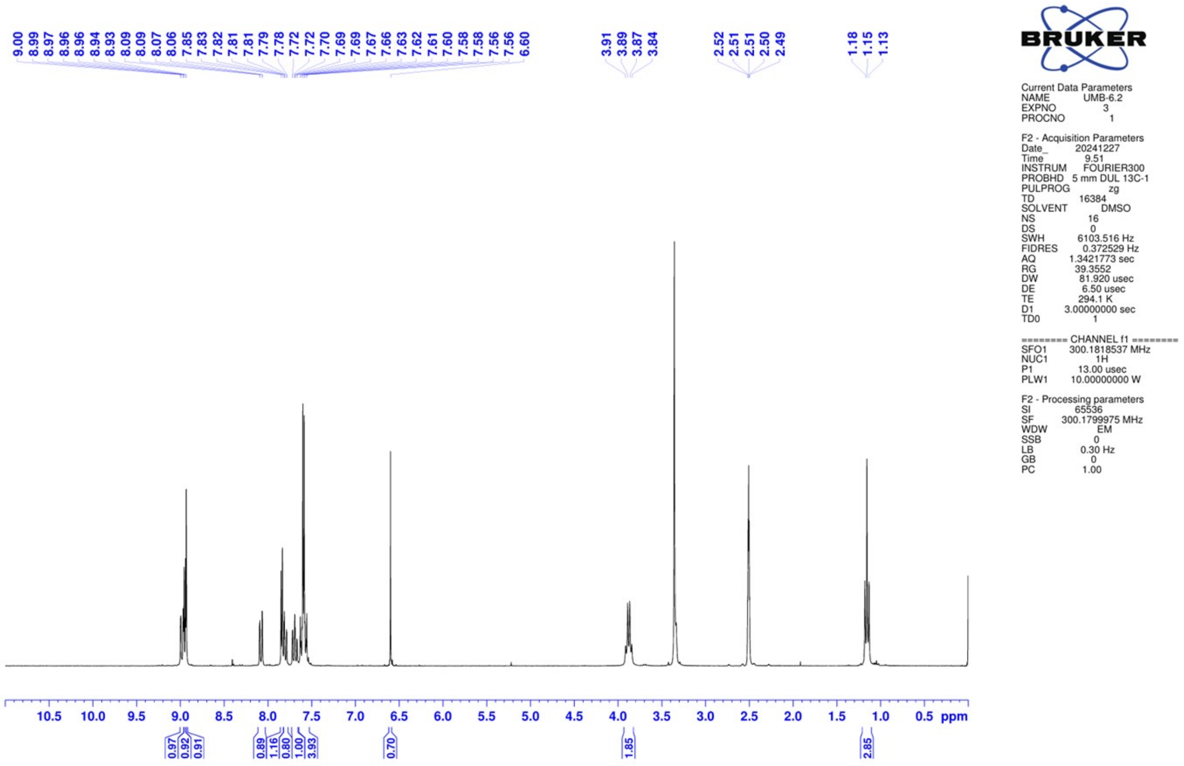

Spectra 16. ^{1}H-NMR spectra of compound **3f**

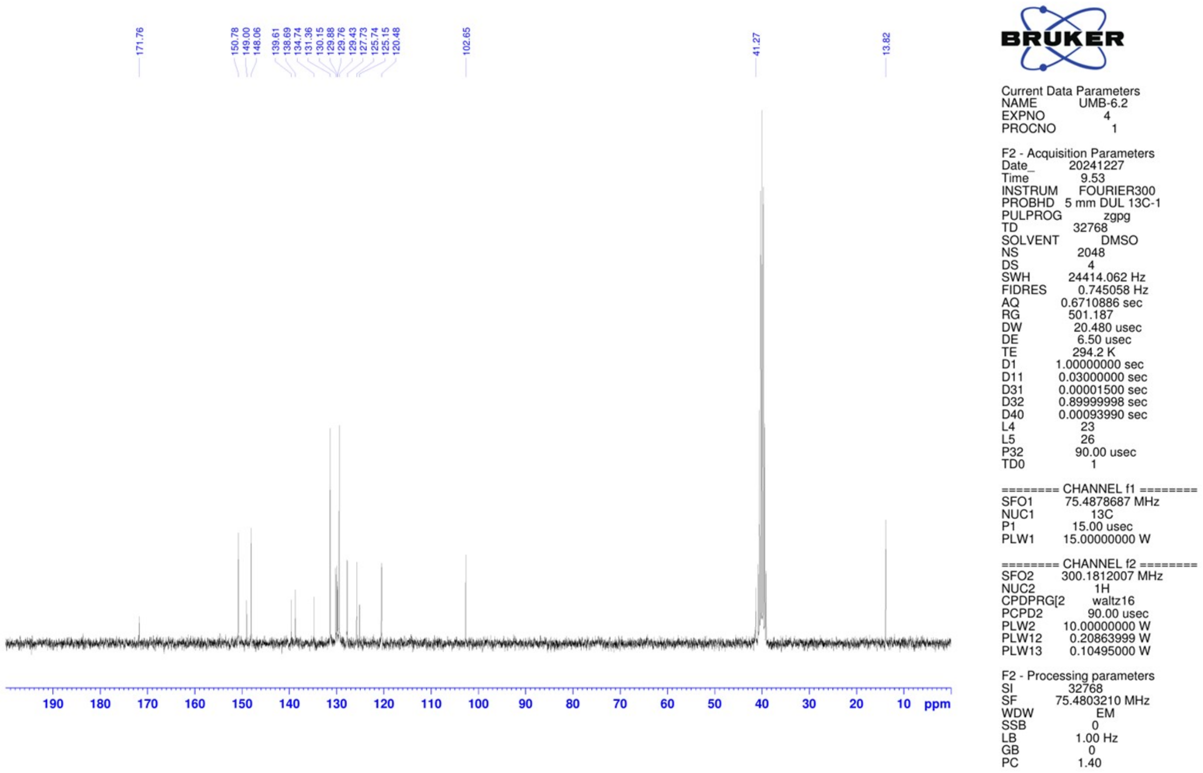

Spectra 17. ^{13}C-NMR spectra of compound **3f**

Data File: C:\LabSolutions\Data\Analiz\Derya\UMB-6.2_86.lcd

Elmt	Val.	Min	Max	Elmt	Val.	Min	Max	Elmt	Val.	Min	Max	Elmt	Val.	Min	Max	Use Adduct
H	1	15	30	O	2	0	0	S	2	1	1	Ru	2	0	0	H
C	4	12	37	F	1	0	0	Cl	1	0	1	Pd	2	0	0	
N	3	1	6	P	3	0	0	Br	1	0	0	I	3	0	0	

Error Margin (ppm):	10	DBE Range:	5.0 - 20.0	Electron Ions:	both
HC Ratio:	unlimited	Apply N Rule:	no	Use MSn Info:	yes
Max Isotopes:	5	Isotope RI (%):	1.00	Isotope Res:	9000
MSn Iso RI (%):	10.00	MSn Logic Mode:	AND	Max Results:	50

Event#: 1 MS(E+) Ret. Time : 1.947 Scan# : 293

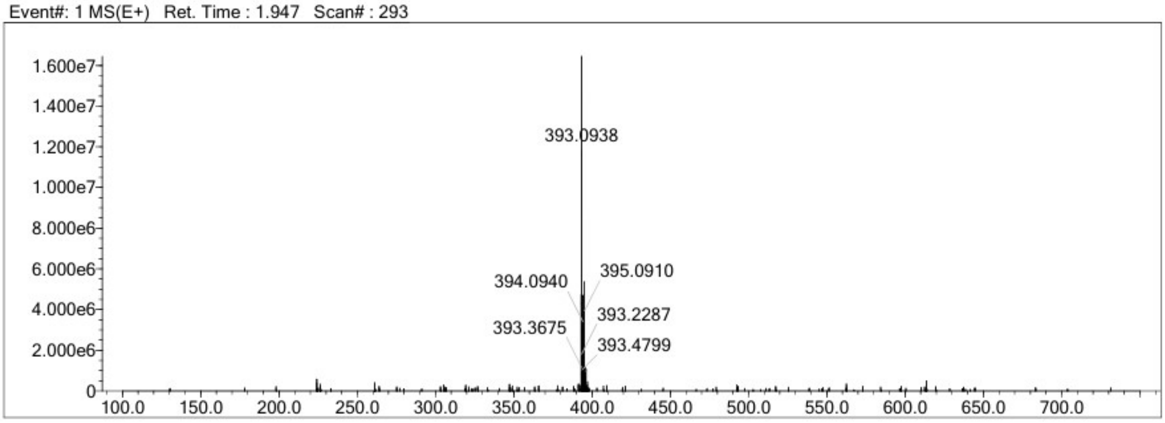

Measured region for 393.0938 m/z

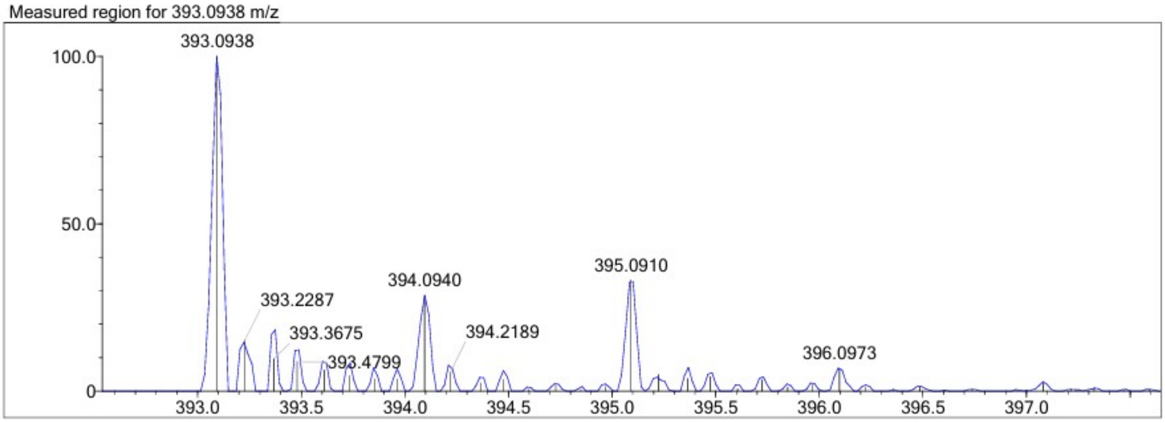

C21 H17 N4 S Cl [M+H]+ : Predicted region for 393.0935 m/z

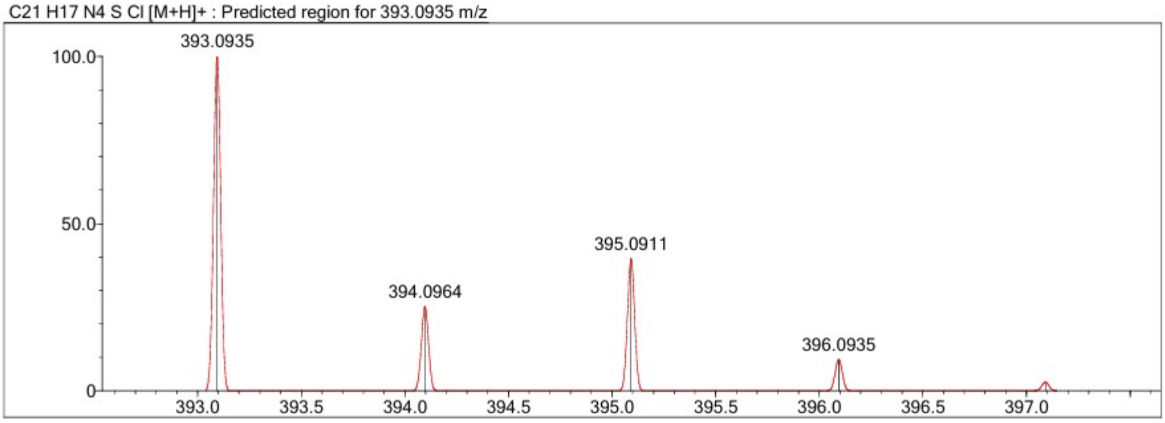

Rank	Score	Formula (M)	Ion	Meas. m/z	Pred. m/z	Df. (mDa)	Df. (ppm)	Iso	DBE
1	68.18	C21 H17 N4 S Cl	[M+H]+	393.0938	393.0935	0.3	0.76	68.18	15.0

Spectra 18. HRMS spectra of compound **3f**

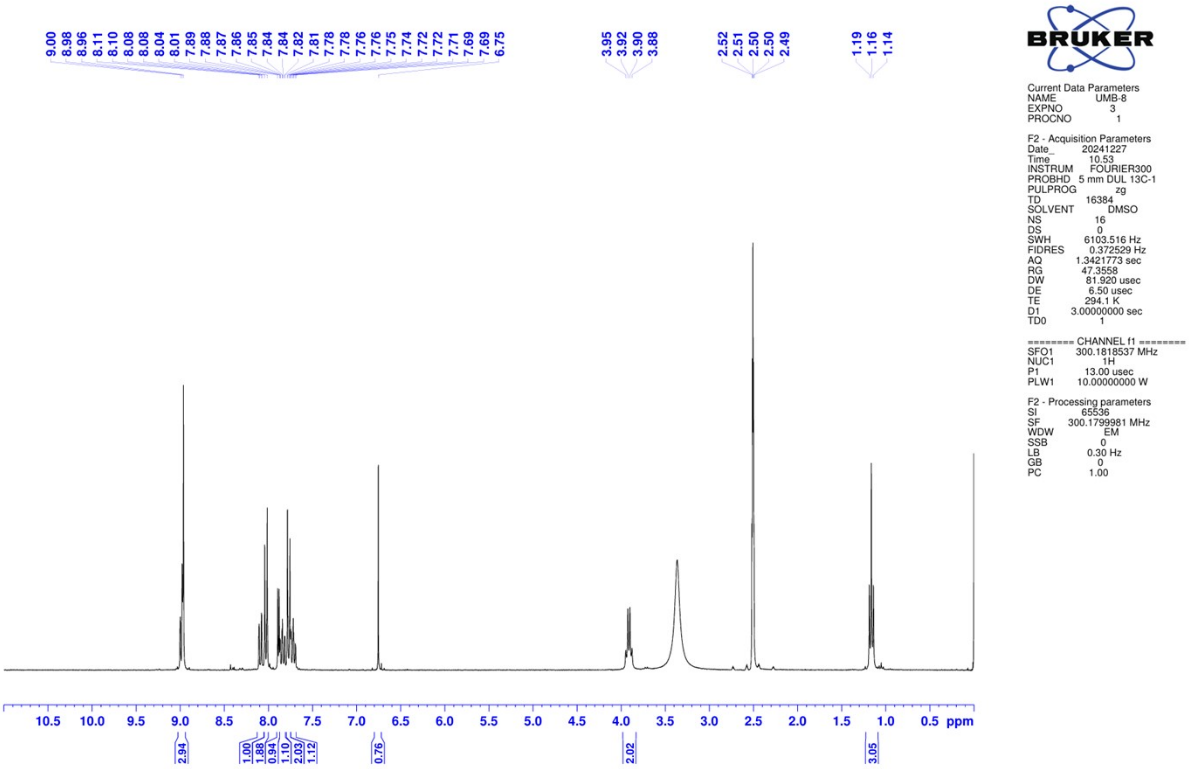

Spectra 19. ^{1}H-NMR spectra of compound **3g**

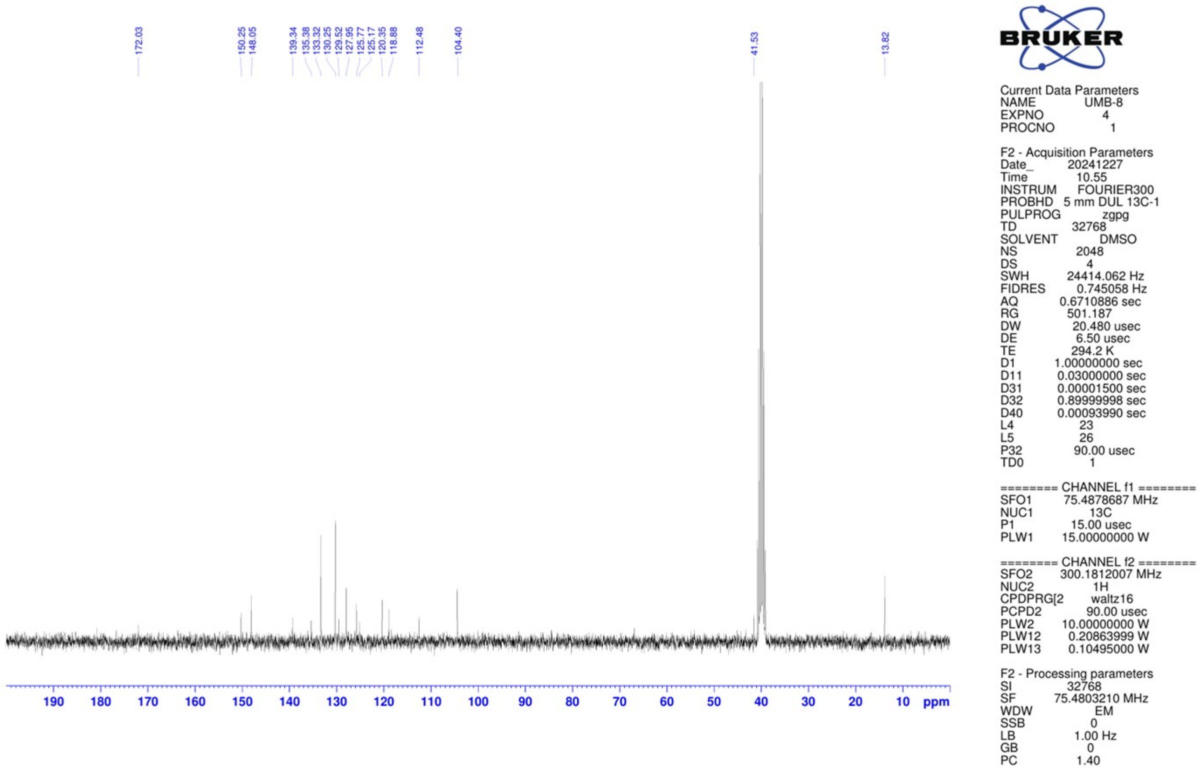

Spectra 20. ^{13}C-NMR spectra of compound **3g**

Formula Predictor Report - UMB-8_27.lcd Page 1 of 1

Data File: C:\LabSolutions\Data\Analiz\Derya\UMB-8_27.lcd

Elmt	Val.	Min	Max	Elmt	Val.	Min	Max	Elmt	Val.	Min	Max	Elmt	Val.	Min	Max	Use Adduct
H	1	5	35	O	2	0	0	S	2	0	2	Ru	2	0	0	H
C	4	9	35	F	1	0	1	Cl	1	0	0	Pd	2	0	0	
N	3	4	8	P	3	0	0	Br	1	0	0	I	3	0	0	

Error Margin (ppm):	5	DBE Range:	8.0 - 30.0	Electron Ions:	both
HC Ratio:	unlimited	Apply N Rule:	yes	Use MSn Info:	yes
Max Isotopes:	3	Isotope RI (%):	1.00	Isotope Res:	9000
MSn Iso RI (%):	10.00	MSn Logic Mode:	AND	Max Results:	50

Event#: 1 MS(E+) Ret. Time : 3.853 Scan# : 579

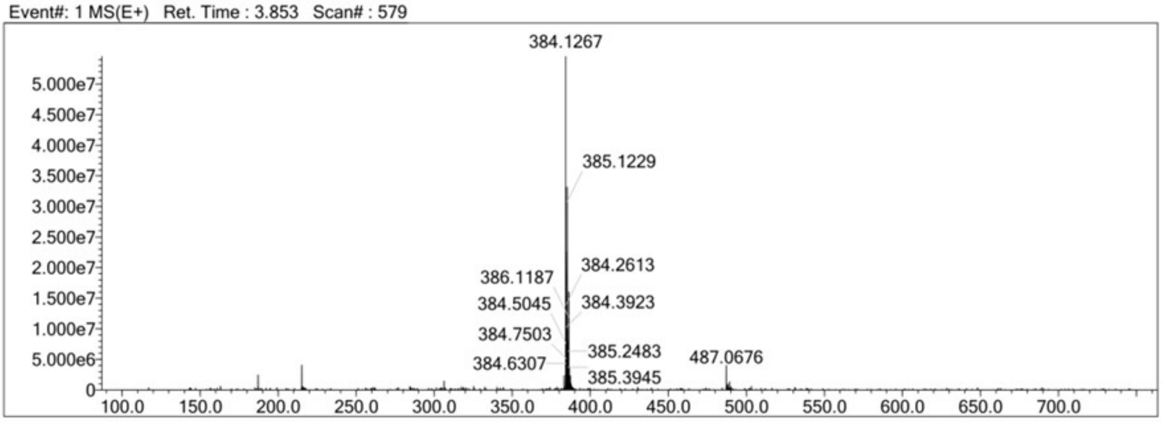

Measured region for 384.1267 m/z

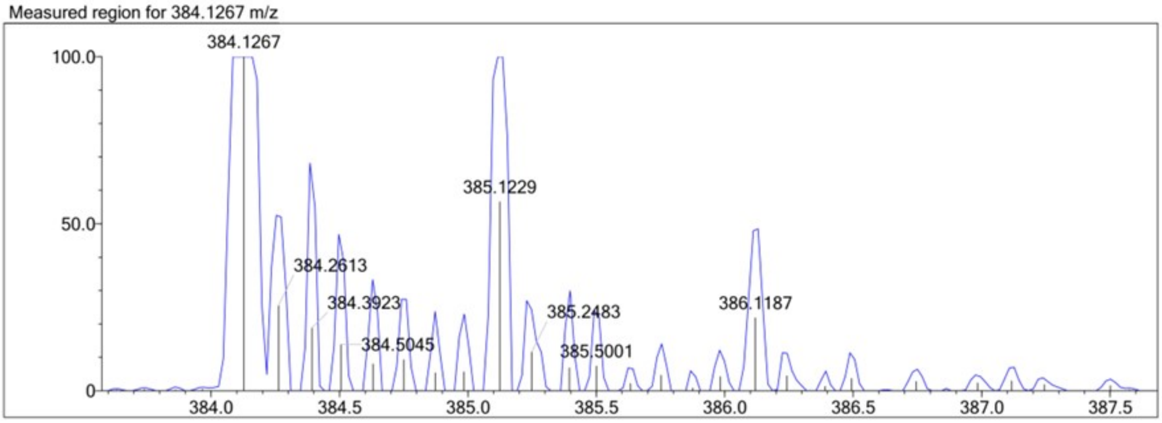

C22 H17 N5 S [M+H]+ : Predicted region for 384.1277 m/z

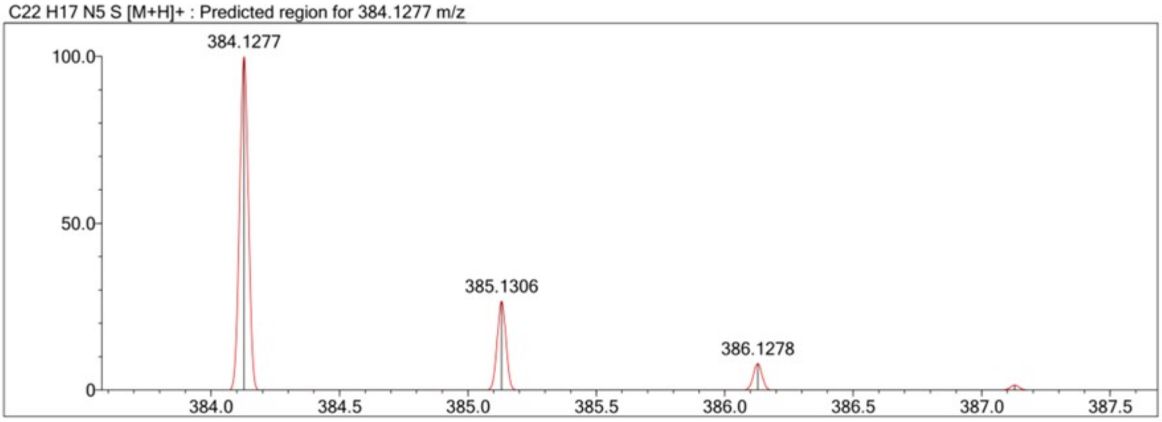

Rank	Score	Formula (M)	Ion	Meas. m/z	Pred. m/z	Df. (mDa)	Df. (ppm)	Iso	DBE
1	21.72	C22 H17 N5 S	[M+H]+	384.1267	384.1277	-1.0	-2.60	22.62	17.0

Spectra 21. HRMS spectra of compound **3g**

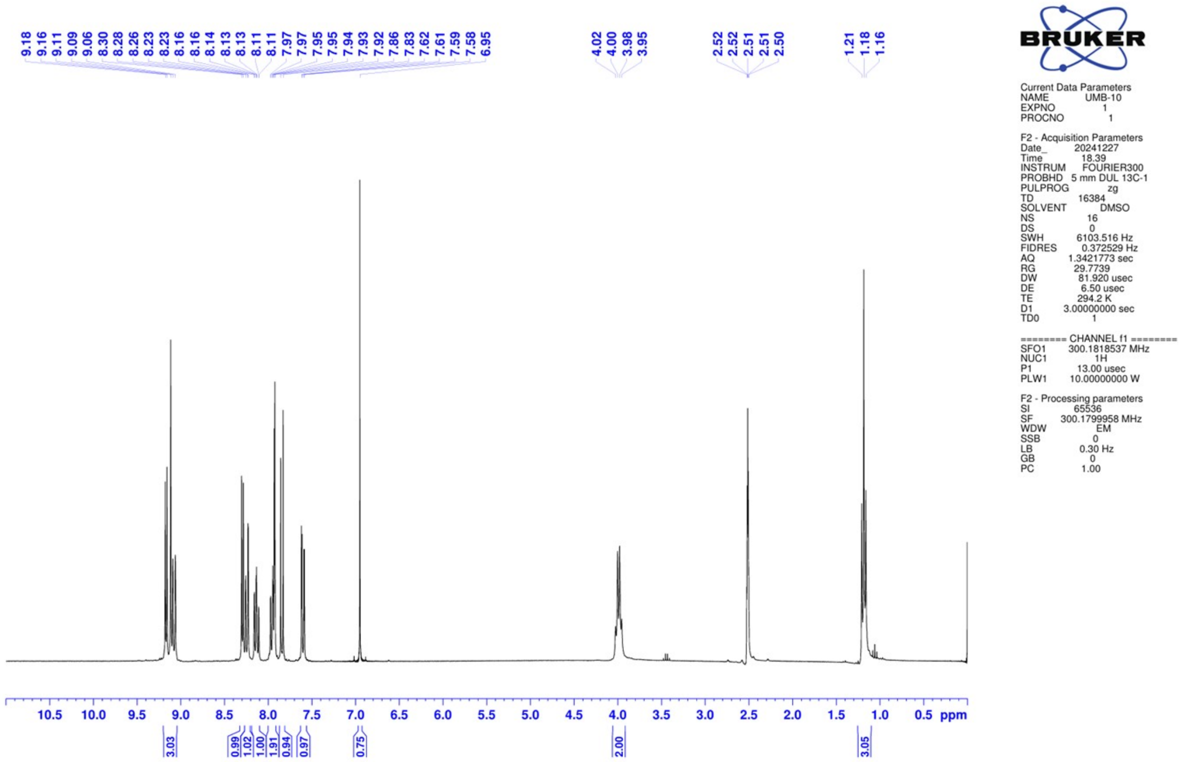

Spectra 22. ^{1}H-NMR spectra of compound **3h**

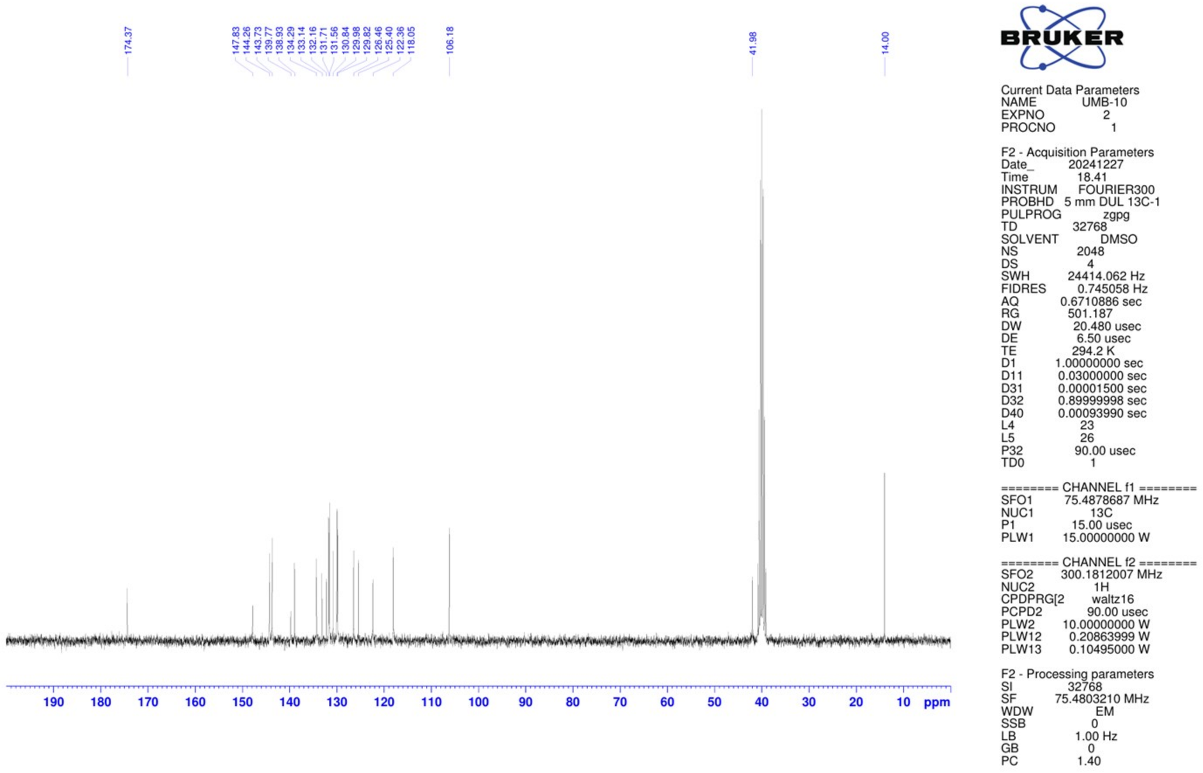

Spectra 23. ^{13}C-NMR spectra of compound **3h**

Formula Predictor Report - UMB-10_29.lcd

Data File: C:\LabSolutions\Data\Analiz\Derya\UMB-10_29.lcd

Elmt	Val.	Min	Max	Elmt	Val.	Min	Max	Elmt	Val.	Min	Max	Elmt	Val.	Min	Max	Use Adduct
H	1	5	35	O	2	0	0	S	2	0	2	Ru	2	0	0	H
C	4	9	35	F	1	0	0	Cl	1	0	2	Pd	2	0	0	
N	3	4	8	P	3	0	0	Br	1	0	0	I	3	0	0	

Error Margin (ppm):	10	DBE Range:	8.0 - 19.0	Electron Ions:	both
HC Ratio:	unlimited	Apply N Rule:	yes	Use MSn Info:	yes
Max Isotopes:	3	Isotope RI (%):	1.00	Isotope Res:	9000
MSn Iso RI (%):	10.00	MSn Logic Mode:	AND	Max Results:	50

Event#: 1 MS(E+) Ret. Time : 7.600 Scan# : 1141

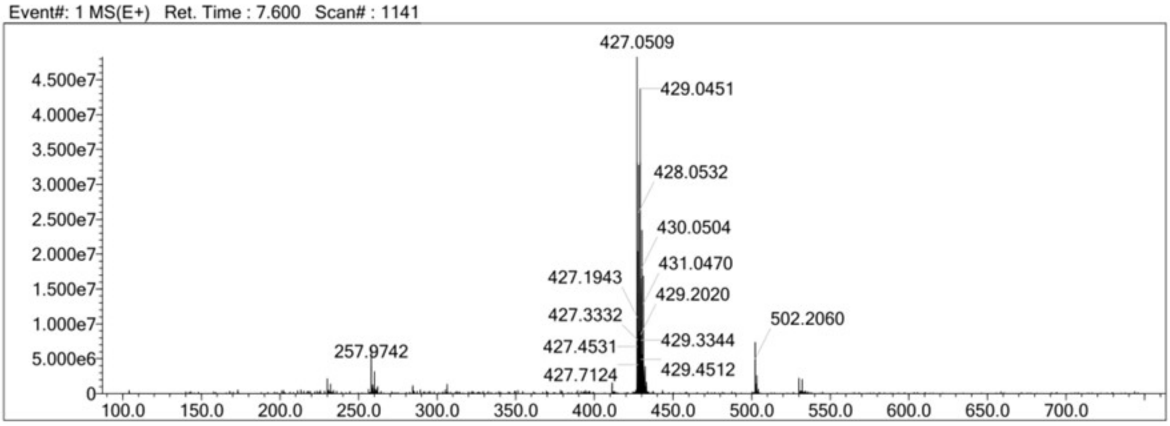

Measured region for 427.0509 m/z

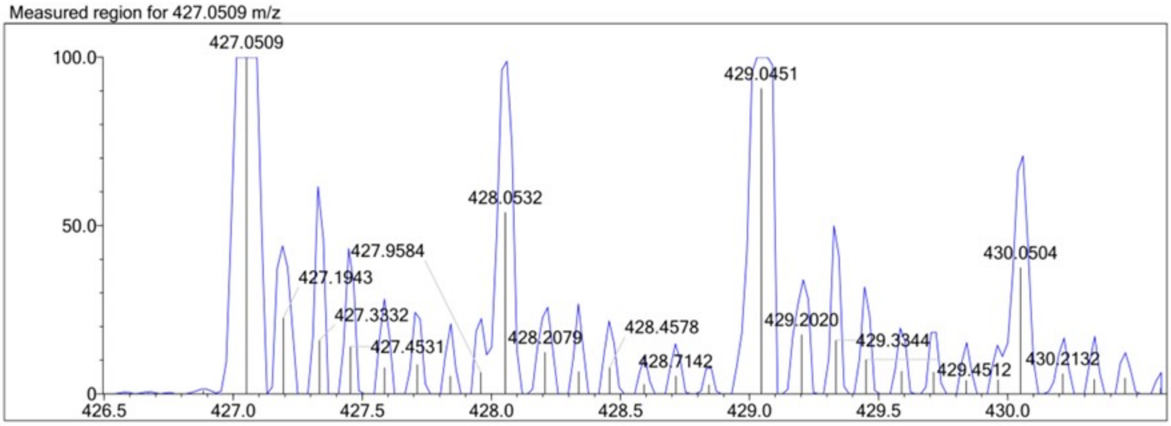

C21 H16 N4 S Cl2 [M+H]+ : Predicted region for 427.0545 m/z

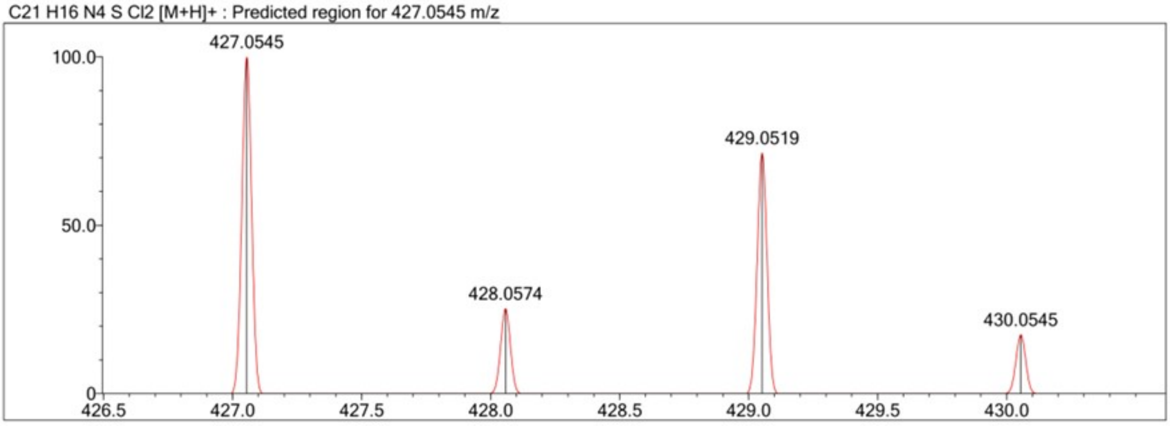

Rank	Score	Formula (M)	Ion	Meas. m/z	Pred. m/z	Df. (mDa)	Df. (ppm)	Iso	DBE
1	20.23	C21 H16 N4 S Cl2	[M+H]+	427.0509	427.0545	-3.6	-8.43	36.31	15.0

Spectra 24. HRMS spectra of compound **3h**

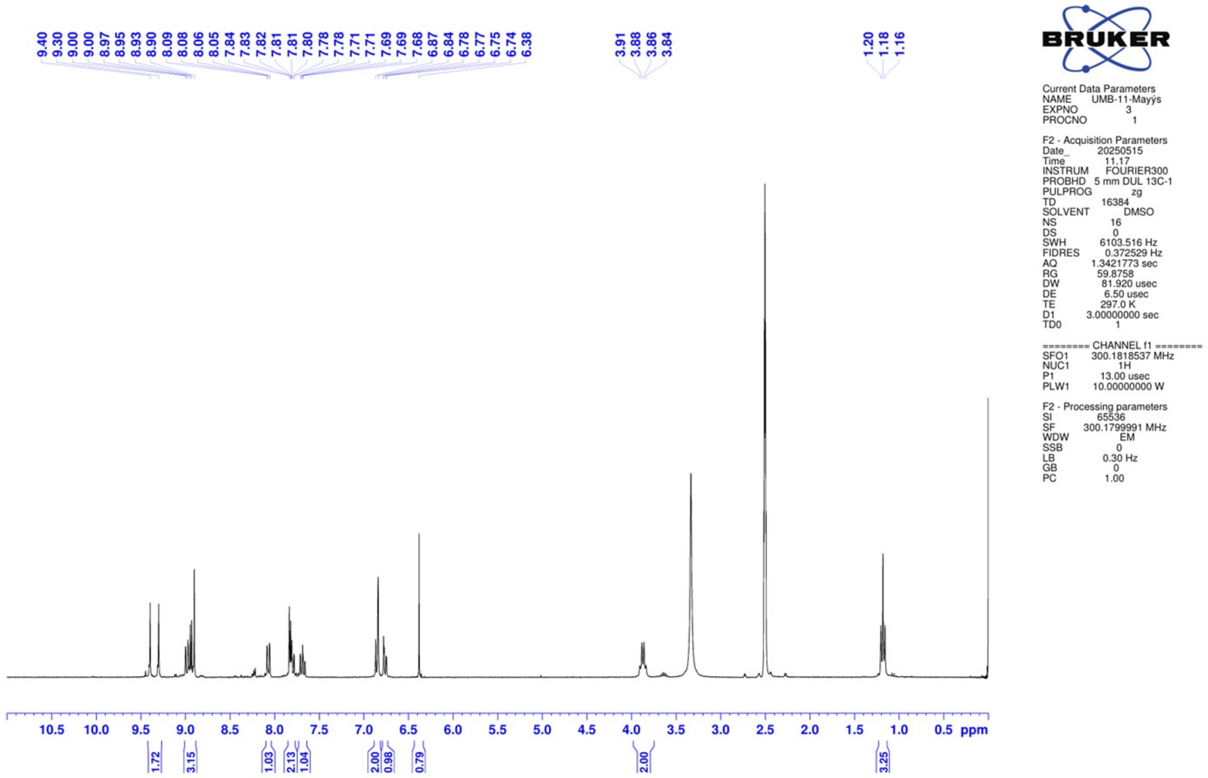

Spectra 25. ^{1}H-NMR spectra of compound **3i**

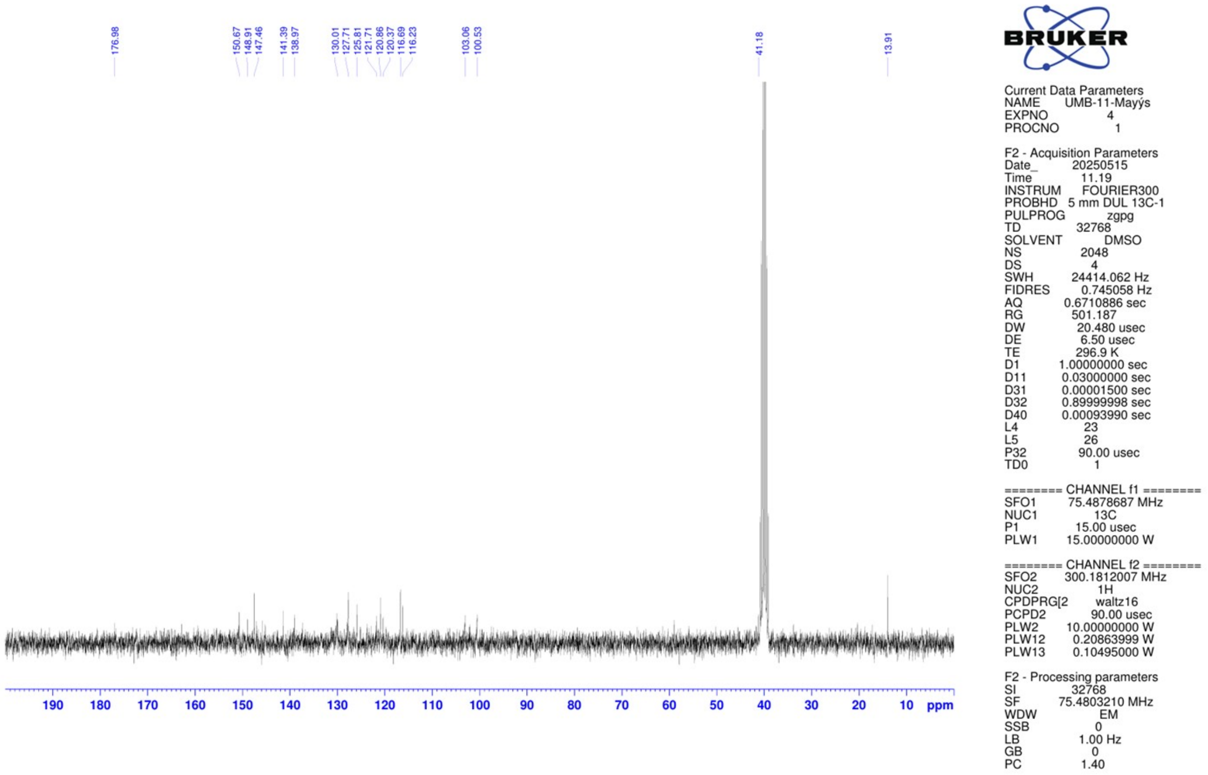

Spectra 26. ^{13}C-NMR spectra of compound **3i**

Data File: C:\LabSolutions\Data\Analiz\Derya\UMB-11_30.lcd

Elmt	Val.	Min	Max	Elmt	Val.	Min	Max	Elmt	Val.	Min	Max	Elmt	Val.	Min	Max	Use Adduct
H	1	5	35	O	2	2	2	S	2	0	2	Ru	2	0	0	H
C	4	9	35	F	1	0	0	Cl	1	0	2	Pd	2	0	0	
N	3	4	8	P	3	0	0	Br	1	0	0	I	3	0	0	

Error Margin (ppm): 5
HC Ratio: unlimited
Max Isotopes: 3
MSn Iso RI (%): 10.00

DBE Range: 8.0 - 19.0
Apply N Rule: yes
Isotope RI (%): 1.00
MSn Logic Mode: AND

Electron Ions: both
Use MSn Info: yes
Isotope Res: 9000
Max Results: 50

Event#: 1 MS(E+) Ret. Time : 2.533 Scan# : 381

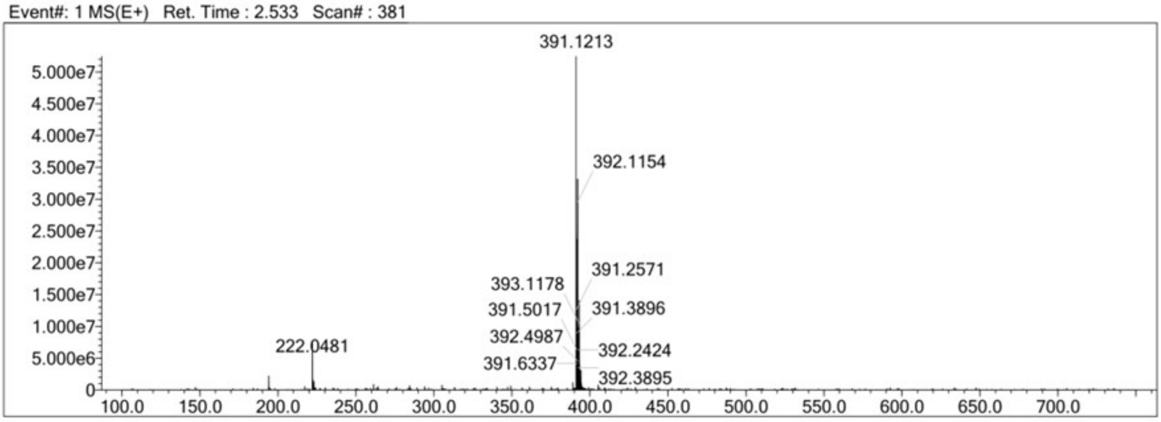

Measured region for 391.1213 m/z

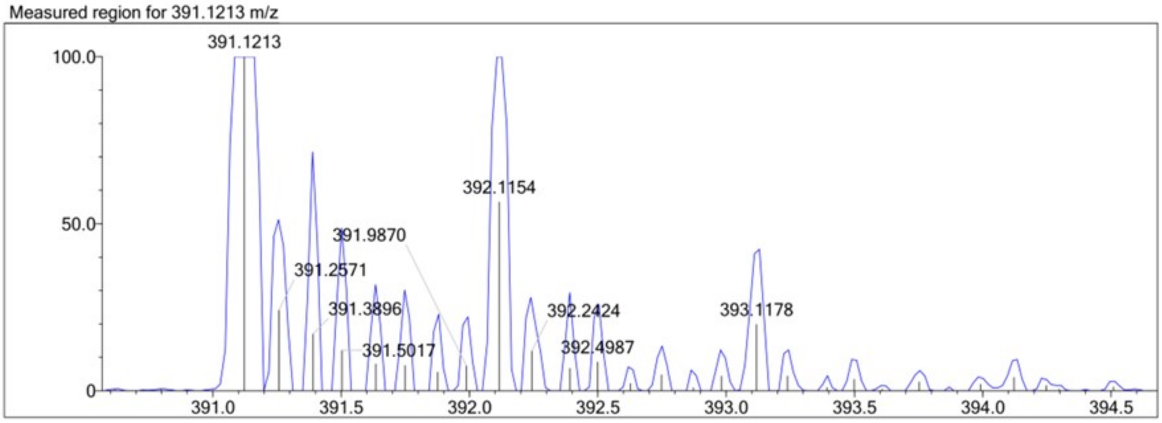

C21 H18 N4 O2 S [M+H]+ : Predicted region for 391.1223 m/z

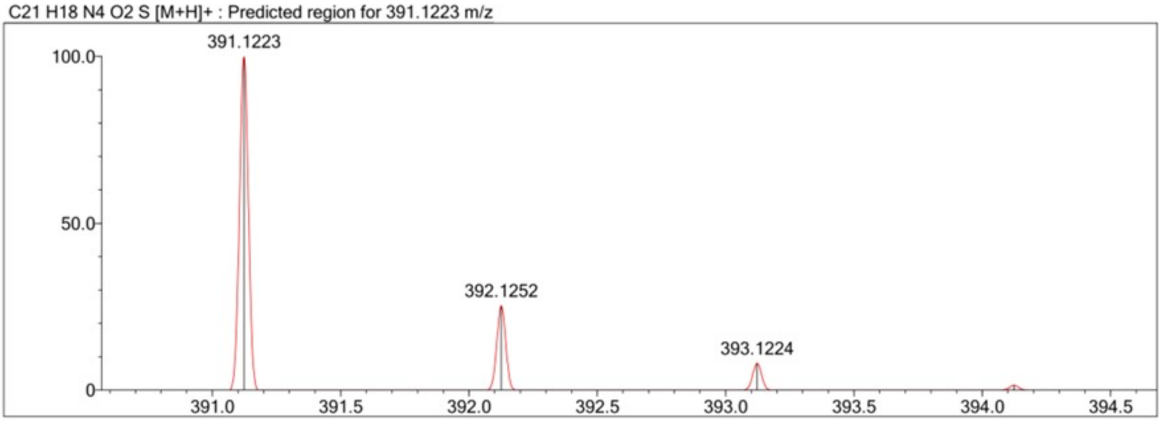

Rank	Score	Formula (M)	Ion	Meas. m/z	Pred. m/z	Df. (mDa)	Df. (ppm)	Iso	DBE
1	30.03	C21 H18 N4 O2 S	[M+H]+	391.1213	391.1223	-1.0	-2.56	31.25	15.0

Spectra 27. HRMS spectra of compound **3i**

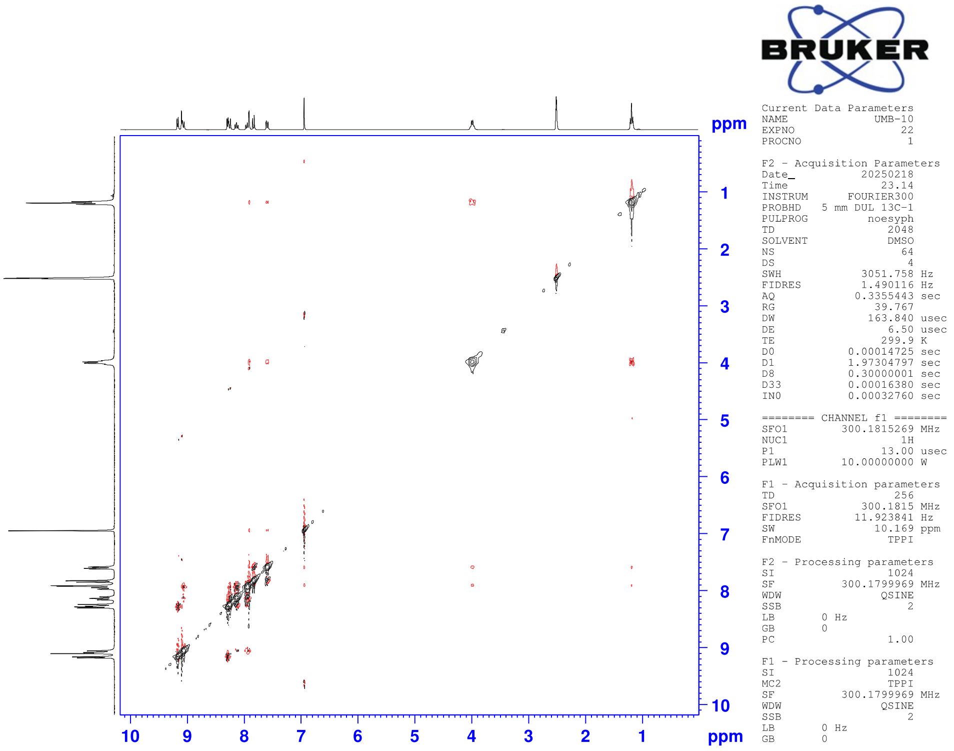

Spectra 28. NOESY spectra of compound **3i**

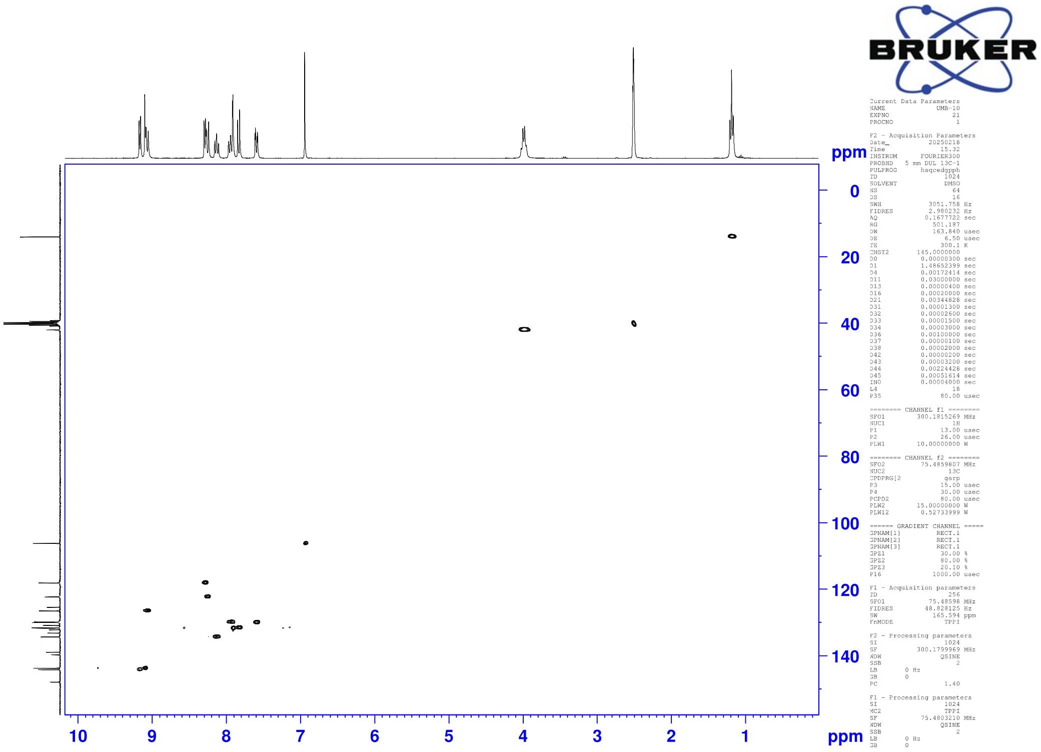

Spectra 29. HSQC spectra of compound **3i**

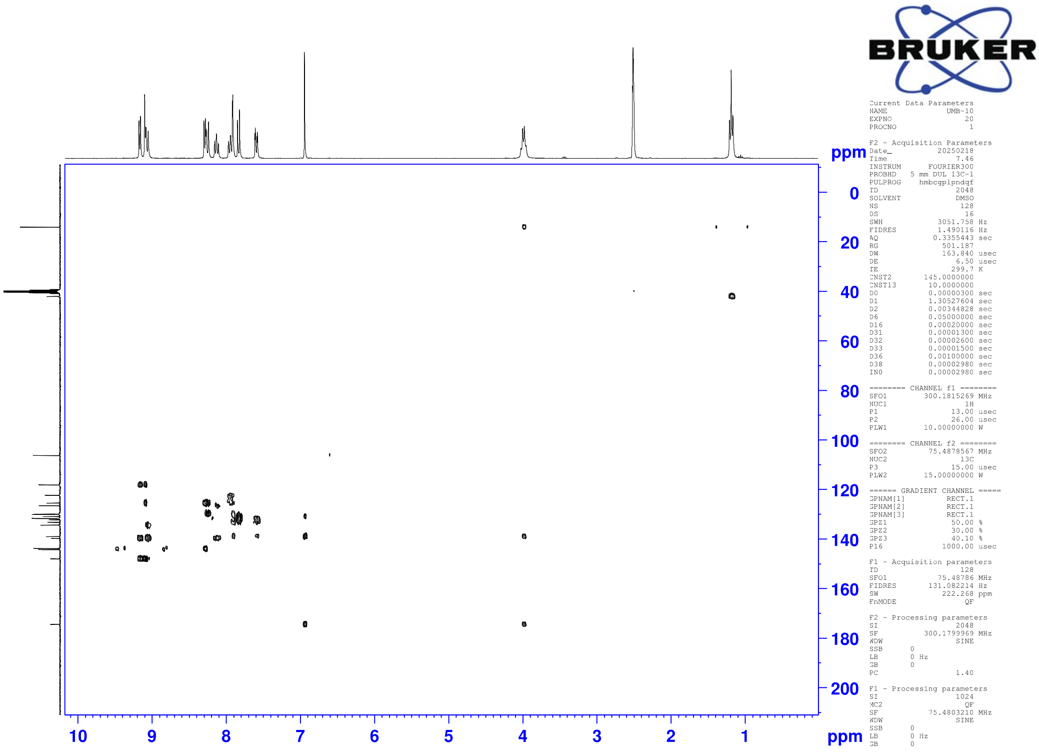

Spectra 30. HMBC spectra of compound **3i**
